# Trace element bioaccumulation in the freshwater mussel *Unio elongatulus* after long-term transplantation: implications for biomonitoring and nature-based remediation

**DOI:** 10.1007/s10653-026-03424-0

**Published:** 2026-08-20

**Authors:** Irene Geneselli, Simone Savi, Sheila Rinaldi, Maria Urbanska, Maria Chiara Fontanella, Gian Maria Beone, Nicoletta Riccardi

**Affiliations:** 1 CNR- Water Research Institute, Viale Tonolli 50, 28922 Verbania Pallanza, VB Italy; 2 Poznań University of Life Sciences, Ul. Wojska Polskiego 28, 60-637 Poznań, Poland; 3 Department for Sustainable Food Process, Università Cattolica del Sacro Cuore, Via Emilia Parmense 84, 29122 Piacenza, PC Italy

**Keywords:** Freshwater mussels, Active biomonitoring, Trace element bioaccumulation, Translocation experiment, Sediment-associated contamination, Shell geochemistry

## Abstract

Freshwater mussels are widely used as bioindicators of aquatic contamination due to their high filtration capacity and ability to accumulate trace elements. However, their potential role in contaminant sequestration and environmental remediation remains poorly explored under natural exposure conditions. In this study, we investigated trace element bioaccumulation dynamics in the freshwater mussel *Unio elongatulus* (Pfeiffer, 1825) through a long-term active biomonitoring experiment. Approximately 2,000 individuals collected from an uncontaminated reference site in Lake Maggiore (Italy) were translocated to Lake Orta, a historically metal-contaminated system, and retrieved after two years of natural exposure. Element concentrations were quantified in sediments, soft tissues and shells of donor, transplanted and resident mussel populations. Sediments showed the strongest spatial differentiation between lakes, reflecting differences in catchment characteristics and long-term geochemical inputs. After prolonged exposure, element concentrations in soft tissues of translocated mussels converged toward those of the resident population, indicating that tissue accumulation primarily reflects current environmental availability rather than population origin. Shell composition displayed clearer spatial differentiation and recorded longer-term exposure patterns. Bioconcentration factors revealed strong accumulation for several elements, including Mn, Sr and Ba, whereas metals such as Fe, Co and Mo showed limited biological uptake. These results demonstrate that *Unio elongatulus* integrates trace element exposure across multiple temporal scales and confirm the value of combining sediment, tissue and shell analyses for environmental assessment. This multi-compartment approach improves the interpretation of element dynamics in freshwater ecosystems and supports the use of freshwater mussels as effective tools for biomonitoring and nature-based remediation of contaminated waters.

**Supplementary Information:**

The online version contains supplementary material available at https://doi.org/10.1007/s10653-026-03424-0.

## Introduction

The preservation of water quality and ecosystem integrity requires effective detection, assessment, and remediation of environmental pollutants. Bioaccumulators are widely used in environmental monitoring because they integrate contaminant exposure over time and reflect the biologically available fraction of pollutants. As a result, they provide valuable information on pollutant persistence and bioavailability, enabling the assessment of past and present contamination and the identification of long-term environmental trends.

Bivalves have long been employed as sentinel organisms due to their capacity to accumulate contaminants in their tissues without immediate adverse effects. Their high filtration rates, strong bioaccumulation potential and often dense populations, which allow the collection of statistically robust replicate samples (Vaughn, 2018), make freshwater bivalves particularly suitable for biomonitoring (e.g. Verma et al., 2025; Yap et al., 2021; Zieritz et al., 2022; Bakshi et al., 2023, Zuykov et al., 2013). Consequently, they are widely used to assess spatial and temporal trends in water quality, identify pollution sources and evaluate the effectiveness of remediation actions or the impacts of contamination events.

Beyond their role in monitoring, freshwater mussels have also been proposed as potential agents for environmental remediation. Their high filtration efficiency, ability to accumulate multiple pollutants and sedentary lifestyle promote localized contaminant sequestration (Zieritz et al., 2022 and references therein; Verma et al., 2025).

Freshwater mussels have been shown to reduce pathogen and nutrient loads in wastewater effluents (e.g. Bianchi et al., 2014; De Franco, 2017; Ferreira-Rodríguez et al., 2024; Julkunen et al., 2024) and natural aquatic systems (Burns et al., 2014; Marroni et al., 2017), as well as to decrease heavy metal concentrations in metal-rich effluents such as acid mine drainage (Aldridge et al., 2014; McFarland et al., 2015).

Nevertheless, despite extensive evidence of their element bioaccumulation capacity in biomonitoring studies, their direct application in bioremediation remains poorly explored and requires validation under environmentally realistic exposure conditions.

In particular, field-based evidence quantifying metal sequestration by freshwater mussels under prolonged natural exposure remains scarce (Zieritz et al., 2022). Freshwater mussels can accumulate a wide range of elements, including essential (e.g. Na, K, Mg, Ca), non-essential (e.g. Co, Sr, Ba) and toxic elements (e.g. As, Hg, Pb), often at concentrations exceeding those dissolved in the surrounding water (Jeffree et al., 1993; Metcalfe-Smith, 1994; Ravera et al., 2003; Piotrowski et al., 2020). As benthic filter feeders, they are exposed not only to contaminants in the water column and suspended particles but also to sediment-associated elements, making them particularly suitable for assessing pollutant uptake under environmentally realistic conditions (Gillis, 2012).

Assessing the suitability of freshwater mussels for bioremediation requires quantifying pollutant uptake and accumulation under environmentally realistic conditions. Bioaccumulation is influenced by pollutant bioavailability as well as biological traits such as species, age, developmental stage, and physiological adaptation (Luoma & Rainbow, 2008; Metcalfe-Smith, 1994; Rainbow, 2002; Wang & Rainbow, 2008).

Although numerous laboratory studies have investigated element uptake, depuration dynamics, and ecotoxicological responses (e.g. Riisgård & Larsen, 2005; Salánki et al., 2003), controlled experiments often involve simplified exposure scenarios that do not adequately represent the variability of natural systems (Vignati et al., 2007). Consequently, extrapolation of laboratory results to field conditions remains uncertain, and the interpretation of bioaccumulation under natural exposure requires appropriate field-based approaches. In this context, field studies commonly estimate bioaccumulation efficiency using the bioconcentration factor (BCF), defined as the ratio between contaminant concentration in the organism and in the surrounding environment (McGeer et al., 2003; Wang & Rainbow, 2008). However, environmental samples are typically collected simultaneously with organisms and therefore may not accurately represent the exposure conditions experienced throughout the organism’s lifespan. This limitation is particularly relevant for long-lived organisms such as freshwater mussels, for which exposure duration is often unknown and age determination may be uncertain (Haag & Commens-Carson, 2008; Neves & Moyer, 1988).

Active biomonitoring approaches, in which organisms of known origin are transplanted to the study site, can reduce these uncertainties by ensuring a defined exposure history and improving comparability among sites (Smolders et al., 2005). Moreover, comparisons between transplanted and resident organisms may reveal potential local adaptation and help evaluate the pollutant accumulation capacity of the species across different environmental conditions (Luoma & Rainbow, 2008).

In this study, we used an active biomonitoring approach to investigate trace element accumulation in freshwater mussels under environmentally realistic conditions.

To this end, we conducted an active biomonitoring experiment involving the translocation of *Unio elongatulus* from a reference site to a historically metal-contaminated freshwater system. By quantifying trace element concentrations in mussel tissues after a defined field exposure period, we aimed to (i) characterize bioaccumulation under natural field conditions, (ii) assess the efficiency of mussels in sequestering elements from contaminated environments, and (iii) discuss the implications of these findings for freshwater biomonitoring and, more cautiously, for the potential contribution of mussels to contaminant sequestration in polluted freshwater ecosystems. Element concentrations in transplanted mussels were compared with those measured in individuals from the donor population and in resident mussels from the contaminated site in order to distinguish environmentally driven accumulation from site-of-origin effects.

## Methods

### Study design and sampling

A total of 2,000 mussels were collected on May 23rd 2022 from Lake Maggiore (Baveno, 45.914805 N, 8.503632 E), which served as the reference site, within an area of approximately 100 m^2^ at a depth of 5–7 m. Although not a pristine ecosystem, Lake Maggiore has not experienced the severe industrial element contamination that historically affected Lake Orta and is classified as an uncontaminated reference based on long-term contaminant monitoring in mussel tissues (Marziali, 2025). Lake Maggiore also represents the source population for the natural recolonization of Lake Orta. After collection, the mussels were cleaned to remove periphyton and all epibionts, including attached *Dreissena polymorpha*, and transported to the laboratory, where they were screened for internal parasites to prevent the transfer of pathogens or non-native species (Brian et al., 2021), then individually tagged and measured.

On May 25th 2022, the tagged mussels were translocated to Lake Orta (Bagnera di Orta, 45.799646 N, 8.416000 E), a lake historically affected by severe heavy-metal pollution during the twentieth century (Bonacina, 2001 and references therein). Although liming carried out in 1989–1990 restored metal concentrations in the water column to levels comparable to those of Lake Maggiore (Rogora et al., 2016), the sediments of Lake Orta still act as a reservoir of legacy contaminants (Vignati et al., 2016). The mussels were temporarily held in anchored cages near the transplantation site. The following day (May 26th), they were released directly onto the lakebed over an area of approximately 2,000 m^2^ at a depth of about 5 m. Mussels were manually positioned in the sediment in the same orientation typically observed under natural conditions, with the anterior end buried in the sediment and the posterior end exposed to the water column at an angle of approximately 45° relative to the sediment surface.

The experiment was conducted under natural environmental conditions to allow prolonged exposure and reduce biases related to seasonal variability. Physicochemical data for Lake Maggiore and Lake Orta during the study period are reported in the Supplementary Material (Table S1, CNR IRSA, Verbania Branch, 2025). Metal concentrations in soft tissues may vary according to seasonal cycles driven by environmental factors and the physiological status of the organisms (Ravera et al., 2007), whereas shell accumulation reflects longer-term exposure (Wilson et al., 2018). Mussels translocated in 2022 were therefore left in situ for almost 2.5 years (904 days).

Following transplantation, periodic surveys were carried out at approximately 4-month intervals to assess mussel survival, shell growth and possible displacement from the release area. Growth was evaluated on a randomly collected subsample of approximately 50 individuals during each survey. Mussels from the transplanted population, together with specimens from the donor population in Lake Maggiore and the resident population in Lake Orta, were collected on the same sampling date (November 27th 2024) for subsequent analyses.

Element concentrations were measured in mussels from three groups:

 (i) the donor population from Lake Maggiore (Baveno),

 (ii) the translocated population in Lake Orta (Bagnera di Orta), and.

 (iii) the resident population that naturally recolonized Lake Orta in the early 2000s, located near Gozzano (45.746471 N, 8.436725 E) (Riccardi et al., 2016).

A total of 25 individual mussels were selected for chemical analysis. Twelve individuals were collected at Baveno, representing the contemporary donor-site population; seven tagged translocated individuals were retrieved at Bagnera di Orta; and six resident individuals were collected at Gozzano. Each individual represented an independent biological replicate, and no pooled or composite biological samples were prepared.

A map of the area and the sites referenced above are available in the Supplementary Material (Fig. S1).

To characterize environmental contamination, on 27 November 2024, five independent surface sediment samples were collected at each site by scuba divers using a hand scoop from the upper approximately 10 cm of sediment, at spatially distinct points in close proximity to the mussel populations. The samples were placed in separate sealed bags, maintained under cool conditions during transport, and analysed as independent environmental replicates. Mussels and sediments were transported by the chemical laboratories of the Catholic University of the Sacred Heart (Piacenza, Italy) for analysis. Upon arrival at the laboratory, the sediments, which appeared predominantly sandy upon visual inspection, were air-dried before homogenization and chemical digestion.

### Sample preparation and chemical analyses

Soft tissues and shells from the same individual were separated during dissection, placed in individually labelled containers, and stored at − 20 °C until preparation and chemical analysis. Each mussel was processed individually. Shells were rinsed with deionised water to remove adhering sediments and dried at 45 °C. The periostracum was removed following Merschel and Bau (2015) and K. Zhang et al. (2024). Cleaned shells were ground in an agate mortar and re-dried at 35 °C. Approximately 0.5 g of ground shell material was transferred into acid-cleaned Teflon vessels and digested with 5 mL of ultrapure HNO_3_ (65%) and 1 mL of H_2_O_2_ (30%) using a Mars 5 Express microwave digestion system (CEM, USA). The digestion programme consisted of a power ramp up to 1600 W, with sequential steps of 5, 5, and 10 min at 100, 150, and 200 °C, respectively. After cooling, the digests were diluted to a final volume of 50 mL with ultrapure water and filtered through 0.45 μm nylon membranes.

Soft tissues were prepared following K. Zhang et al. (2024). Samples were rinsed with deionised water, sonicated for 5 min, frozen, and lyophilised at − 50 °C. Approximately 1 g of lyophilised tissue was acidified with 10 mL HNO_3_ (65%) and 2 mL H_2_O_2_ (30%) and digested using the same microwave protocol applied to shell samples.The two biological compartments were subsequently prepared, digested, and analysed separately.

Upon arrival at the laboratory, sediment samples were air-dried, manually disaggregated and homogenised using a mortar and pestle, and passed through a 2-mm sieve. The fraction smaller than 2 mm was used for chemical analysis. The sediments appeared predominantly sandy upon visual inspection, although no formal grain-size analysis was performed. A 0.5 g aliquot of each independent sediment sample was digested with aqua regia, consisting of 7.5 mL HCl (37%) and 2.5 mL HNO_3_ (65%), at 95 °C for 2 h using a DIGIPREP block digestion system (SCP Science, USA).

After cooling, digests were diluted to 50 mL with ultrapure water (resistivity > 18 MΩ cm; PURELAB, ELGA, UK), filtered through 0.45 μm nylon membranes (DigiFILTER, SCP Science). All reagents were of analytical grade or higher purity. Shell and soft-tissue samples from each individual mussel were digested and analysed separately.

Major and trace elements (Na, Ca, K, Mg, Fe, Li, Se, P, Ba, V, Sb, Rb, Mo, Co, Mn, Sr, Al, Pb, Zn, Cr, Cd, Cu, Ni, As, Hg) were quantified using ICP-OES (5100, Agilent Technologies, Santa Clara, CA, USA) for more abundant elements and ICP-MS (7800, Agilent Technologies, Santa Clara, CA, USA) equipped with a High Matrix Introduction (HMI), aerosol-dilution device to reduce the total sample load entering the plasma, for elements occurring at lower concentrations. Each digest was measured in triplicate, and the instrumental replicates were averaged to obtain one concentration value for each individual and biological compartment. Instrumental replicates were not treated as independent biological observations in the statistical analyses.

Calibration standards were prepared from a commercial multi-element stock solution and certified single-element stock solutions. The multi-element standard was PlasmaCAL SCP28AES (SCP Science), containing 100 mg L^−1^ of each element in 5% HNO_3_. Separate single-element stock solutions were used for Hg (MSHG-100PPM, Inorganic Ventures; 99.83 ± 0.48 mg L^−1^ in 10% HCl), K (CGK1, Inorganic Ventures; 998 ± 5 mg L^−1^ in 0.1% HNO_3_), P (CGP10-1, Inorganic Ventures; 9997 ± 43 mg L^−1^ in H_2_O), and Rb (P/N S4400-1000451, Peak Performance; 1000 ± 3 mg L^−1^ in 2% HNO_3_). Intermediate and working calibration solutions were prepared by serial dilution with ultrapure water and adjusted to match the acid matrix of the digested samples. Five calibration levels were used. Element-specific calibration ranges and coefficients of determination are reported in Supplementary Table S4a.

Analytical quality assurance and quality control included procedural digestion blanks, certified reference materials, duplicate sample digestions, and triplicate instrumental measurements. The IAEA-407 fish tissue and BCR-280R lake sediment reference materials were analysed in triplicate. Procedural blanks were included in each analytical batch and processed using the same reagents and digestion procedures as the corresponding samples. Independent duplicate digestions were performed for 10% of the total samples to assess analytical repeatability. Agreement between duplicate digestions was evaluated using the relative percentage difference, and an RPD below 20% was adopted as the acceptance criterion. Method detection limits were calculated as three times the standard deviation of six procedural blanks prepared and diluted in the same manner as the samples (Spalla et al., 2009). Recommended or certified concentrations, concentrations measured in the present study, recoveries, and relative standard deviations obtained for the reference materials are reported in Supplementary Table S4b.

Instrumental operating conditions are reported in the Supplementary Material (Table S2 and S3), and quality control procedures, including certified reference material performance, are fully described in the Table S4a and S4b.

### Statistical analysis

Statistical analyses were performed separately for sediments, soft tissues, and shells. For mussel samples, the biological sample sizes were n = 12 for Baveno, n = 7 for Bagnera di Orta, and n = 6 for Gozzano. Each individual mussel represented one independent biological replicate. Soft-tissue and shell concentrations were obtained from the same individuals butwere analyzed separately by compartment. For sediments, five independent environmental replicates were analyzed at each site (n = 5). Instrumental measurements were performed in triplicate and averaged before statistical analysis; technical replicates were not treated as independent observations.

Data were assessed for normality and homogeneity of variance using the Shapiro–Wilk test and Levene’s test. When assumptions were met, one-way analysis of variance (ANOVA) followed by Tukey’s post-hoc test was applied. When parametric assumptions were not satisfied, even after transformation, the non-parametric Kruskal–Wallis test was used, followed by Dunn’s post-hoc test. Principal Component Analysis (PCA) was performed separately for sediments, shells, and soft tissues to explore patterns of variability and relationships among elements. Sediment-based bioconcentration factors (BCFs) were calculated as the ratio between the concentration of each element in mussel tissues (shell or soft tissue) and its concentration in the sediment. BCF values > 1 were interpreted as indicating relative enrichment in mussel tissues compared with the surrounding environment. Daily accumulation rates were calculated to evaluate how element concentrations in translocated mussels approached those measured in the resident population.

The daily net change in element concentration was calculated as the difference between the mean concentration measured in transplanted mussels after exposure and that measured in the donor population, divided by the 904-day exposure period. This metric represents the average daily change in whole-compartment concentration and not the rate of elemental incorporation into newly formed shell carbonate. The daily net change in shell element concentration should not be interpreted as the rate of elemental incorporation into newly deposited shell carbonate, but rather as the average temporal change in the elemental concentration of the whole shell over the exposure period.

Mercury (Hg) concentrations were statistically compared only in mussel tissues and shells because sediment concentrations were below detection limits. Phosphorus (P) and barium (Ba) were not analyzed in soft tissues and were therefore excluded from graphical representations.

All statistical analyses were performed using GraphPad Prism 10.6.1.

## Results

**Fig. 1:**
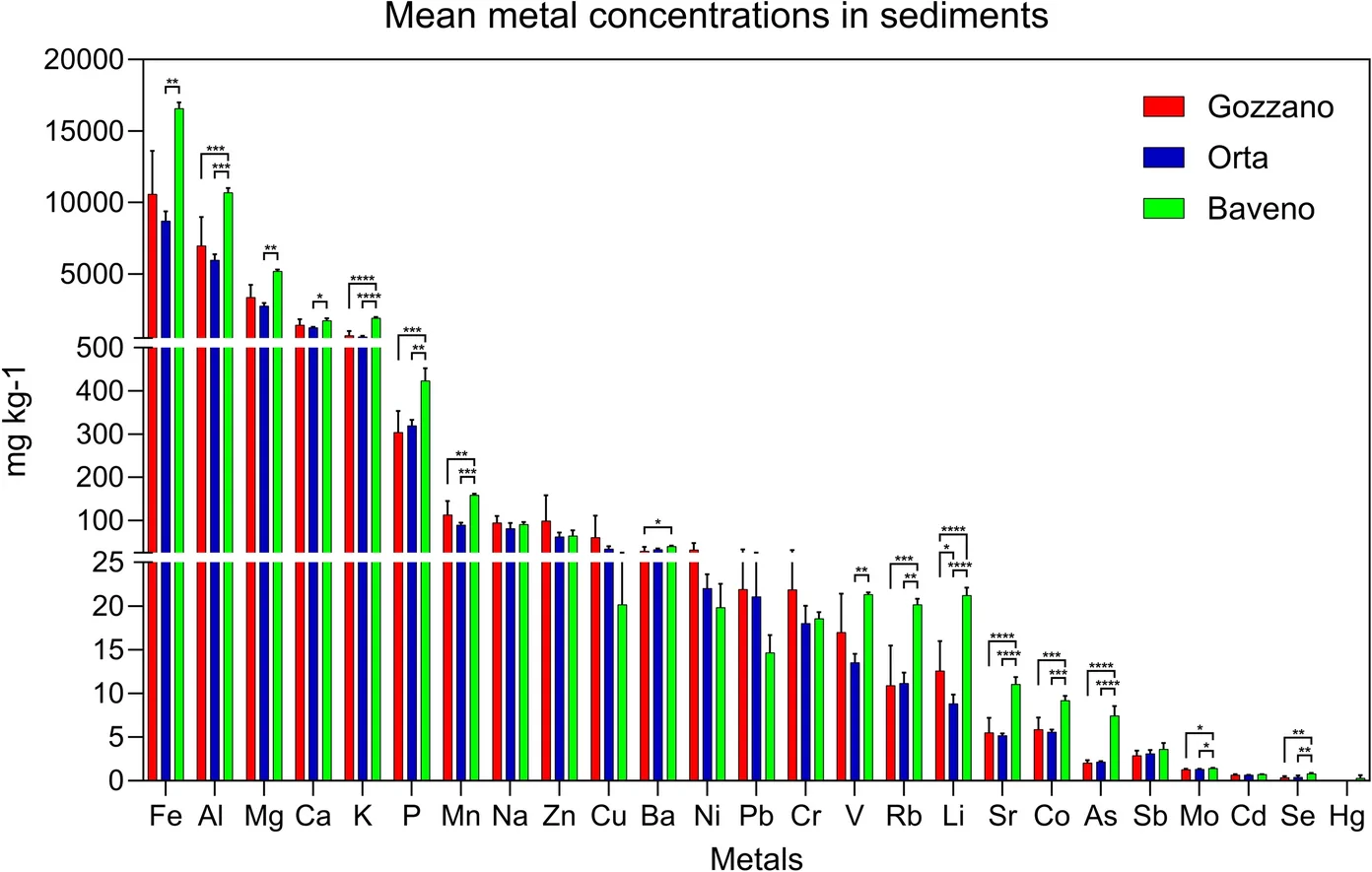
Element concentrations (mg kg^−1^) in sediments collected at three sampling sites: Gozzano (red), Orta (blue) and Baveno (green). Values are expressed as mean ± standard deviation. Significant differences between sites are indicated with asterisks, following statistical analysis and post-hoc tests to identify significantly different comparisons between means (* = *p* < 0.05; ** = *p* < 0.01; *** = *p* < 0.001; **** = *p* < 0.0001)

Element concentrations in sediments from the three sampling sites are summarized in Fig. 1, while detailed values are reported in Supplementary Table S5.

Mussels collected from the three sites had a mean shell length of 6.23 ± 0.82 cm and were analyzed for element concentrations in both soft tissues and shells.

Figures 3 and 6 show the mean concentrations only for elements that exhibited a statistically significant difference among sampling sites in at least one of the two compartments (soft tissues or shells). Elements showing no significant site-related differences in either compartment are not included for clarity.

The complete elemental profiles of soft tissues and shells are presented in Supplementary Tables S6 and S7, respectively, while the complete summary of *p*-values for each compartment and element is presented in Table S8. Finally, the relative abundance of elements across sediments, soft tissues and shells is summarized in Supplementary Table S16.

### Sediments

Element concentrations in sediments showed marked spatial variability among sites (Fig. 1). For 16 out of 25 analyzed elements, post-hoc comparisons (Table S9) revealed significantly higher concentrations in Lake Maggiore (Baveno) than in one or both Lake Orta sites (Gozzano and Orta), which were generally similar except for lithium.

**Fig. 2:**
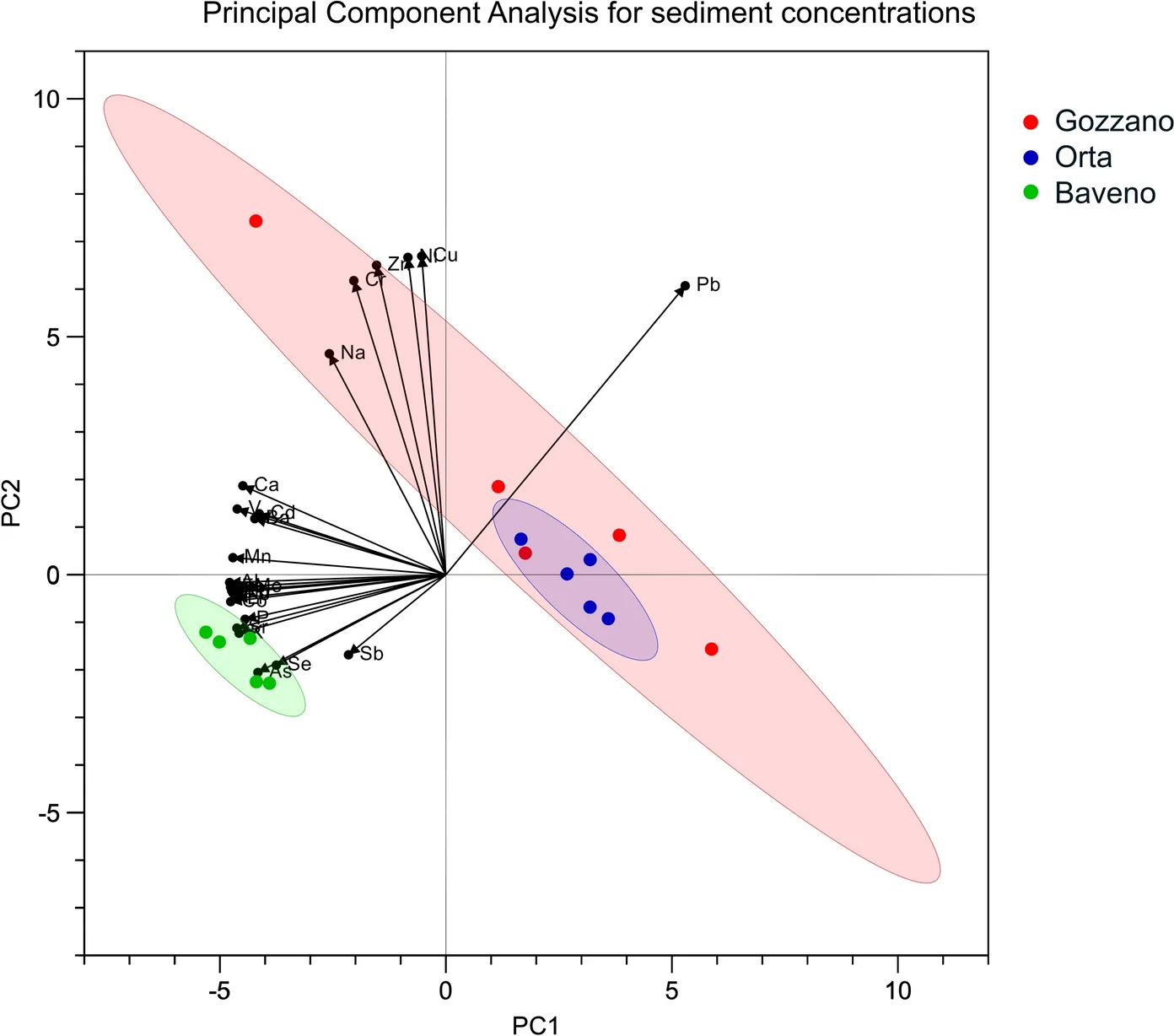
PCA biplot of samples from Gozzano (red), Orta (blue), and Baveno (green) showing clustering by site and loadings of elements measured in sediments. Arrows indicate variable loadings on PC1 and PC2. Colored ellipses represent the 95% confidence intervals for each group

The PCA performed on sediment samples (Fig. 2) confirmed a clear separation between sites belonging to the two lakes. Lake Orta sites clustered together and were distinctly separated from Lake Maggiore along the first principal component. The first two principal components explained 87.70% of the total variability (Table S10).

### Soft tissues

**Fig. 3:**
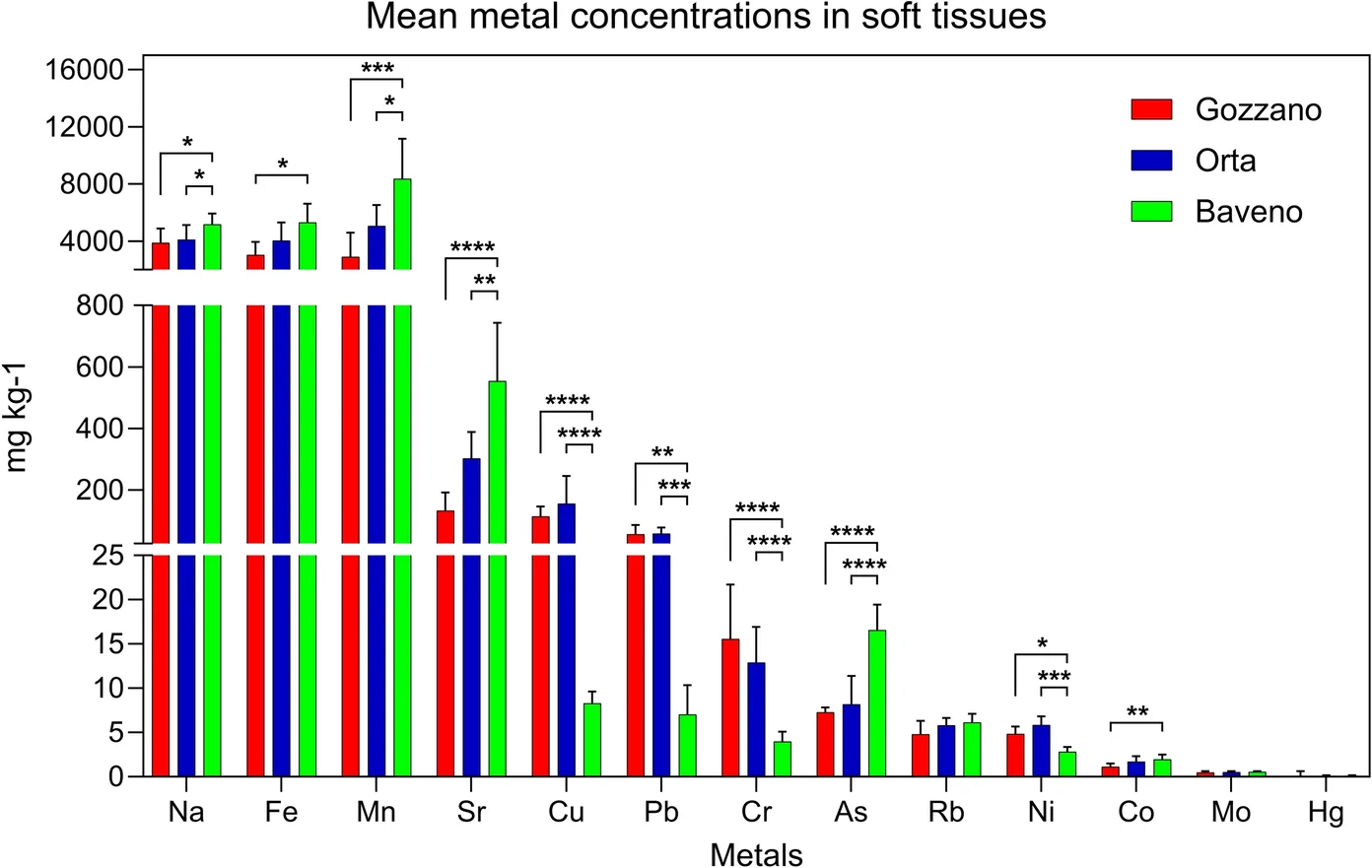
Element concentrations (mg kg^−1^) in soft tissues collected at three sampling sites: Gozzano (red), Orta (blue) and Baveno (green). Values are expressed as mean ± standard deviation. Significant differences between sites are indicated with asterisks, following statistical analysis and post-hoc tests to identify significantly different comparisons between means (* = *p* < 0.05; ** = *p* < 0.01; *** = *p* < 0.001; **** = *p* < 0.0001)

Significant differences among sites were observed for ten elements in soft tissues (Na, Fe, Mn, Sr, Cu, Pb, Cr, As, Ni, Co; Fig. 3), whereas the two Lake Orta sites showed similar concentrations. Post-hoc comparisons revealed two main patterns (Table S11). Copper (Cu), lead (Pb), chromium (Cr) and nickel (Ni) exhibited higher concentrations in Lake Orta than in Lake Maggiore, whereas sodium (Na), manganese (Mn), strontium (Sr) and arsenic (As) showed the opposite trend, with higher concentrations in Lake Maggiore. These patterns were broadly consistent with those observed in sediments.

**Fig. 4:**
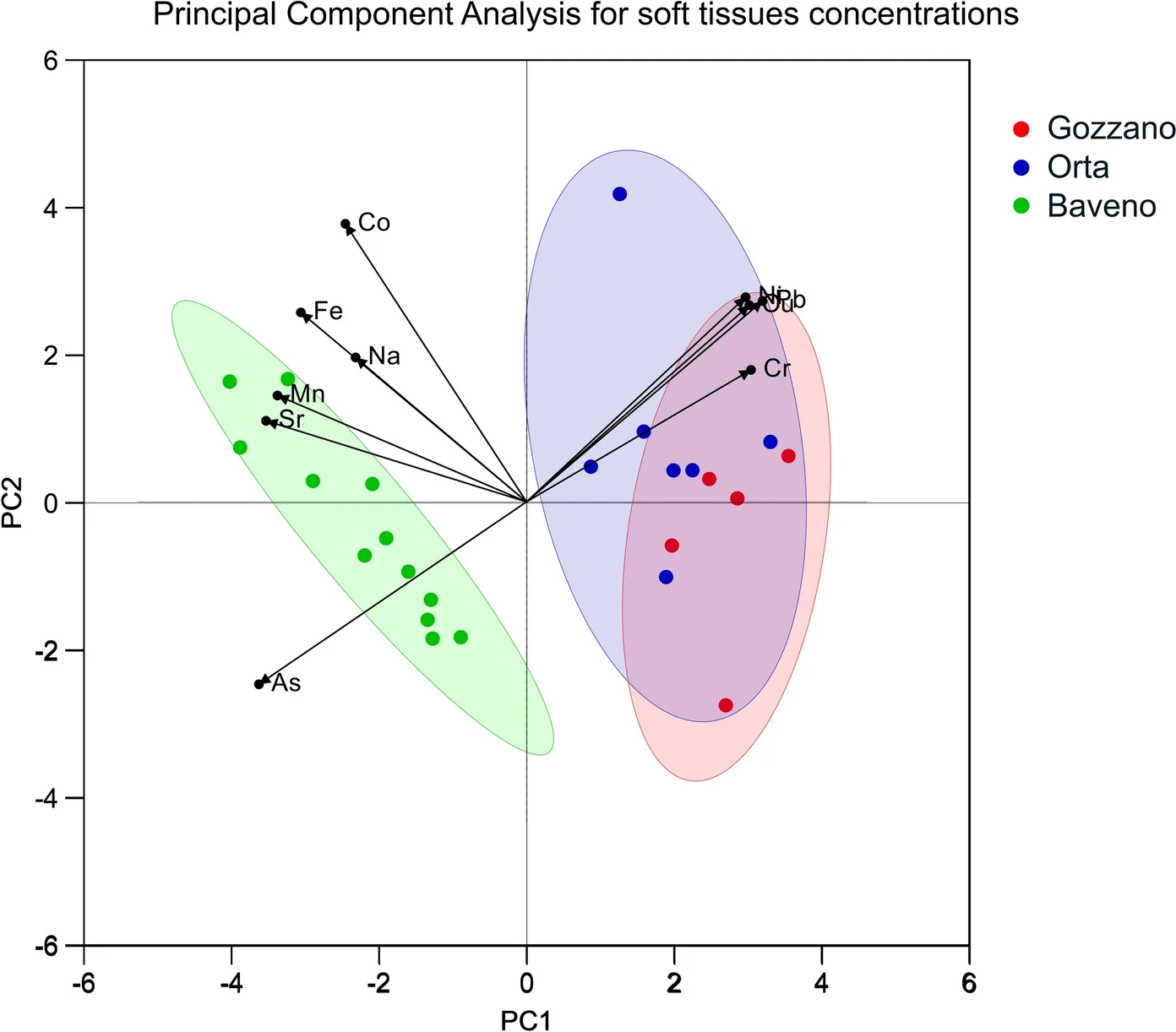
PCA biplot of samples from Gozzano (red), Orta (blue), and Baveno (green) showing clustering by site and loadings of elements measured in tissues. Arrows indicate variable loadings on PC1 and PC2. Colored ellipses represent the 95% confidence intervals for each group

The PCA of soft tissues (Fig. 4) showed weaker spatial separation than sediments, with partial overlap between the two Lake Orta sites. The first two principal components explained 80.81% of the total variability (Table S12).

**Fig. 5:**
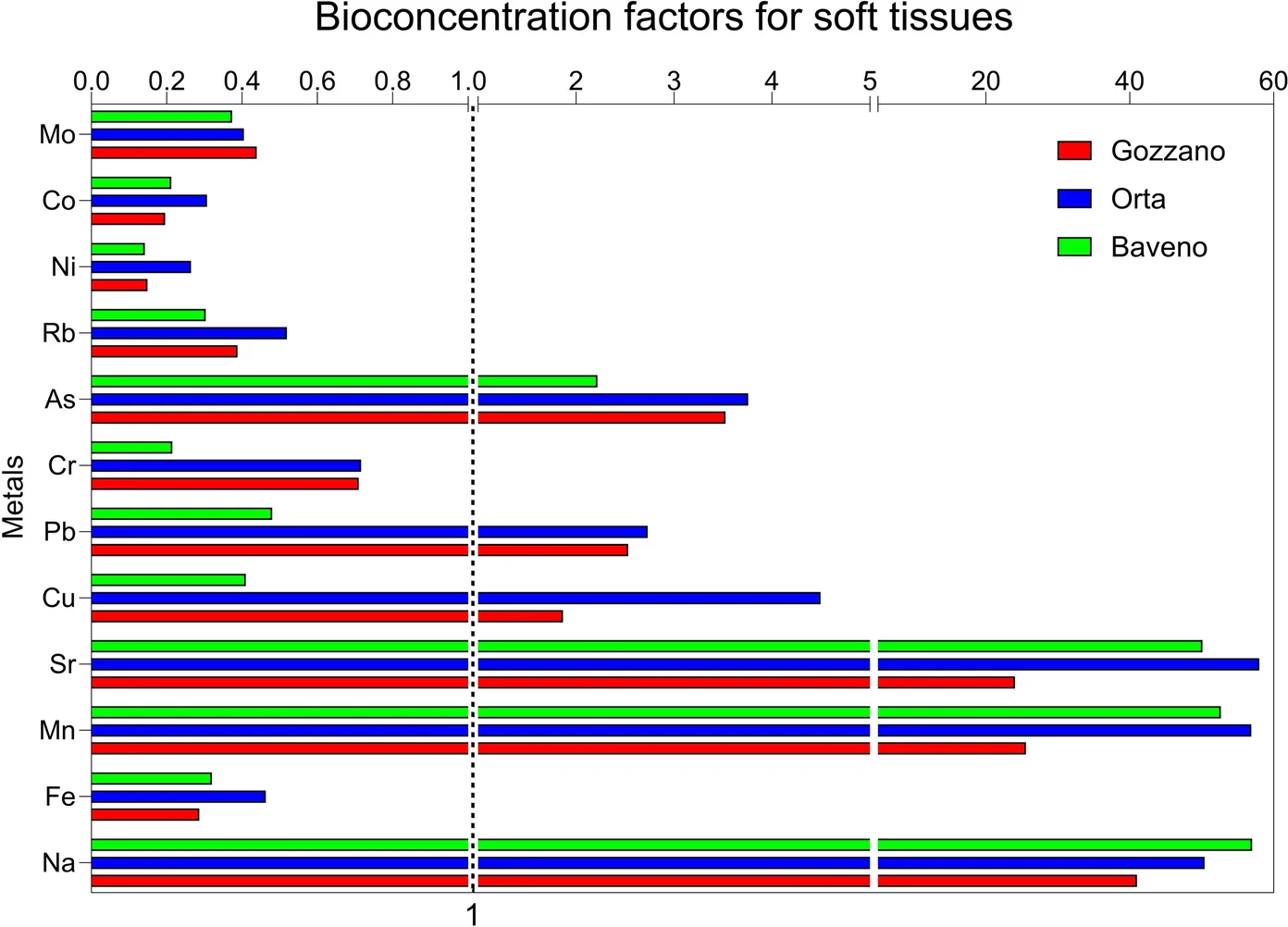
Bioconcentration factor of elements in biological tissues at the three sampling sites. BCF values are reported for each element and site; the y-axis includes multiple breaks to allow the simultaneous visualization of elements spanning different orders of magnitude

BCF in soft tissues are shown in Fig. 5. Overall, BCF values were generally higher at the Orta site (translocated mussels) than at the Gozzano site (resident population), except for molybdenum, which remained very low. Arsenic (As), manganese (Mn), sodium (Na), and strontium (Sr) exhibited BCF values greater than 1 at all sites, while copper (Cu) and lead (Pb) were bioconcentrated only at the Orta site (Table S13).

### Shells

**Fig. 6:**
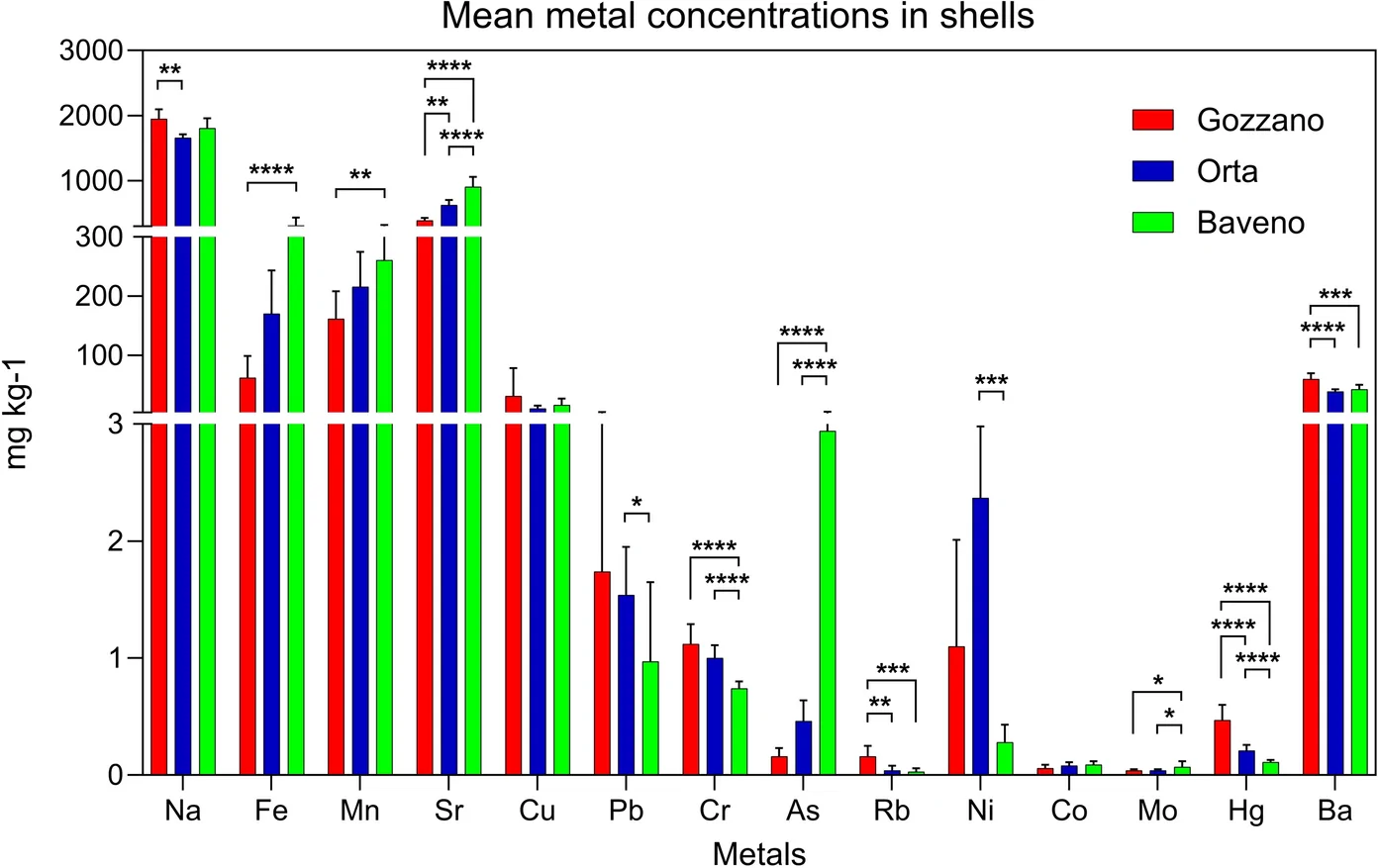
Element concentrations (mg kg^−1^) in shells collected at three sampling sites: Gozzano (red), Orta (blue) and Baveno (green). Values are expressed as mean ± standard deviation. Significant differences between sites are indicated with asterisks, following statistical analysis and post-hoc tests to identify significantly different comparisons between means (* = *p* < 0.05; ** = *p* < 0.01; *** = *p* < 0.001; **** = *p* < 0.0001)

Element concentrations in shells also showed significant spatial variability (Table S14). Significant site-related differences were detected for Na, Fe, Mn, Sr, Pb, Cr, As, Rb, Ni, Mo, Hg and Ba (Fig. 6). As, Cr and Mo consistently differed between Lake Maggiore and Lake Orta sites. Fe, Mn, Ni and Pb differed between Baveno and only one of the Lake Orta sites. Within Lake Orta, Ba, Rb and Na differed between sites, whereas Sr and Hg differed among all three sites.

As showed particularly pronounced differences, with concentrations at Baveno exceeding those at both Lake Orta sites by more than sixfold. Sr and Hg also displayed clear gradients among sites: Sr followed the sequence Baveno > Orta > Gozzano, whereas Hg showed the opposite trend (Gozzano > Orta > Baveno). In addition, Ba, Cr and Rb were consistently higher in Lake Orta than in Lake Maggiore.

**Fig. 7:**
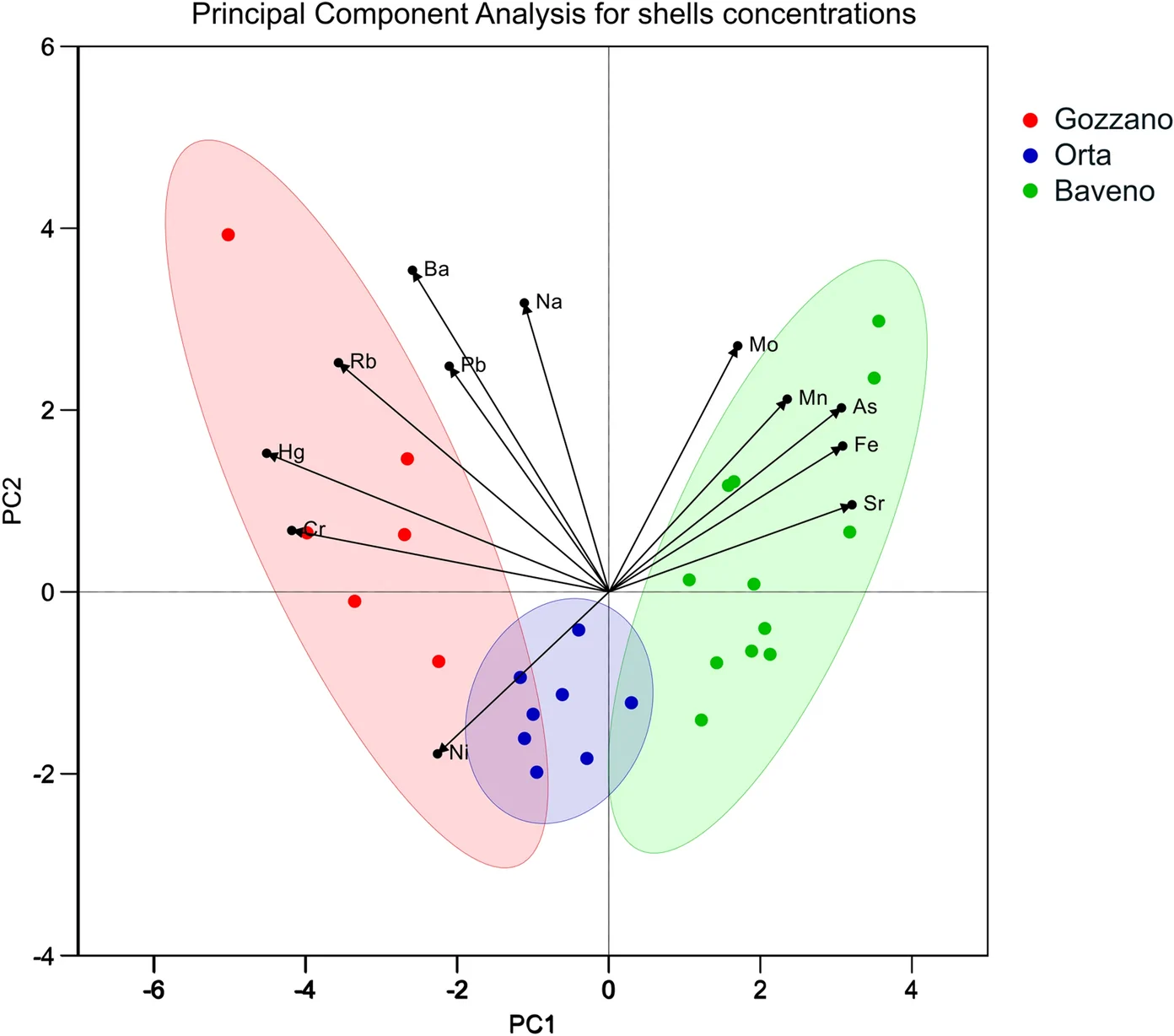
PCA biplot of samples from Gozzano (red), Orta (blue) and Baveno (green) showing clustering by site and loadings of elements measured in shells. Arrows indicate variable loadings on PC1 and PC2. Colored ellipses represent the 95% confidence intervals for each group

The PCA of shells (Fig. 7) revealed clearer separation among the three sites compared with soft tissues, with the translocated population (Orta) positioned between the resident population (Gozzano) and the donor population (Baveno). The first two principal components explained 64.64% of the variability (Table S15).

**Fig. 8:**
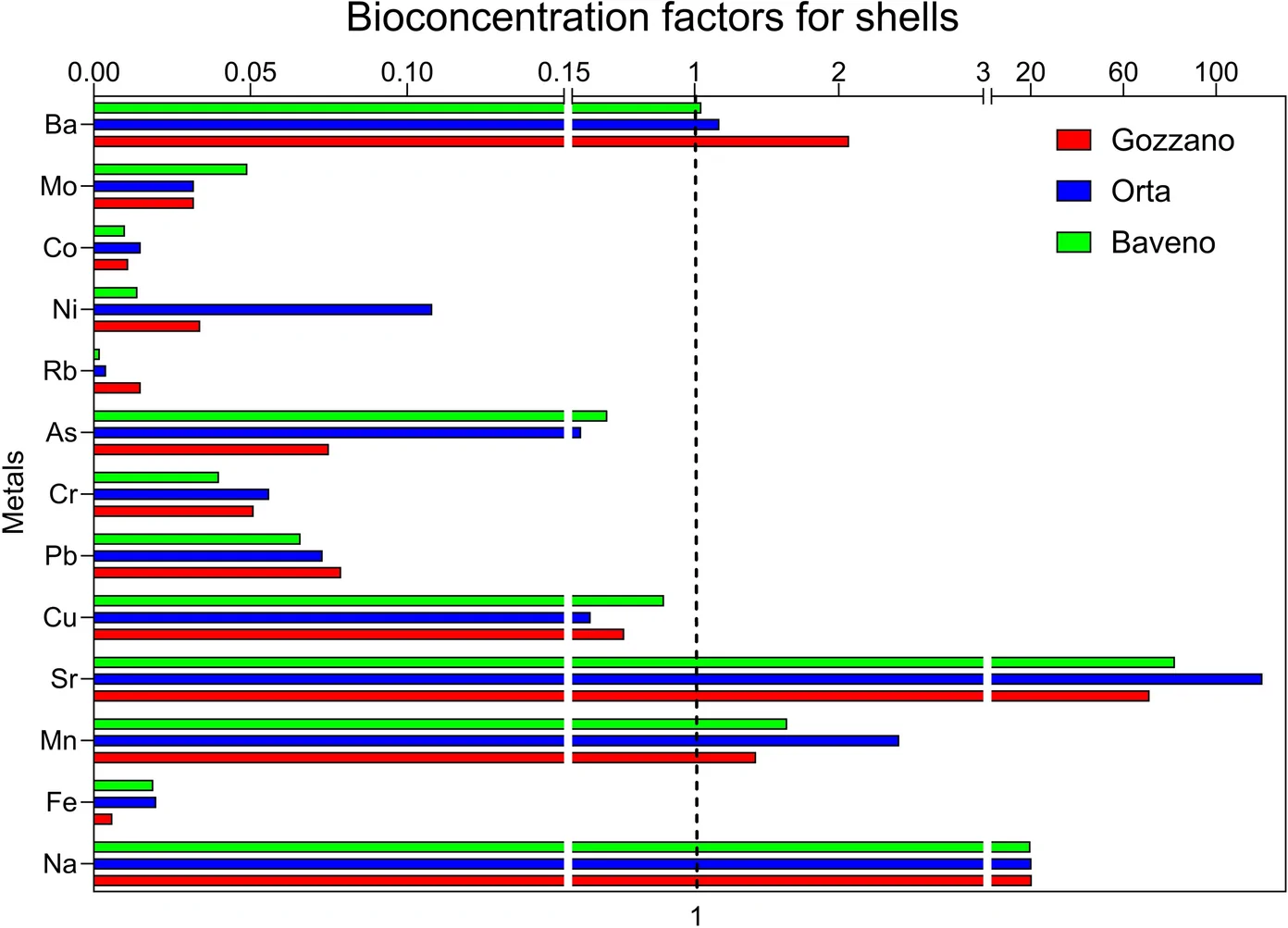
Bioconcentration factor of elements in shells at the three sampling sites. BCF values are reported for each element and site; the y-axis includes multiple breaks to allow the simultaneous visualization of elements spanning different orders of magnitude

BCFs calculated for shells (Fig. 8) showed more heterogeneous patterns than those observed for soft tissues. Ba, Mn, Na and Sr were bioconcentrated (BCF > 1) at all sites (Table S13).

### Net changes in element concentrations

**Fig. 9:**
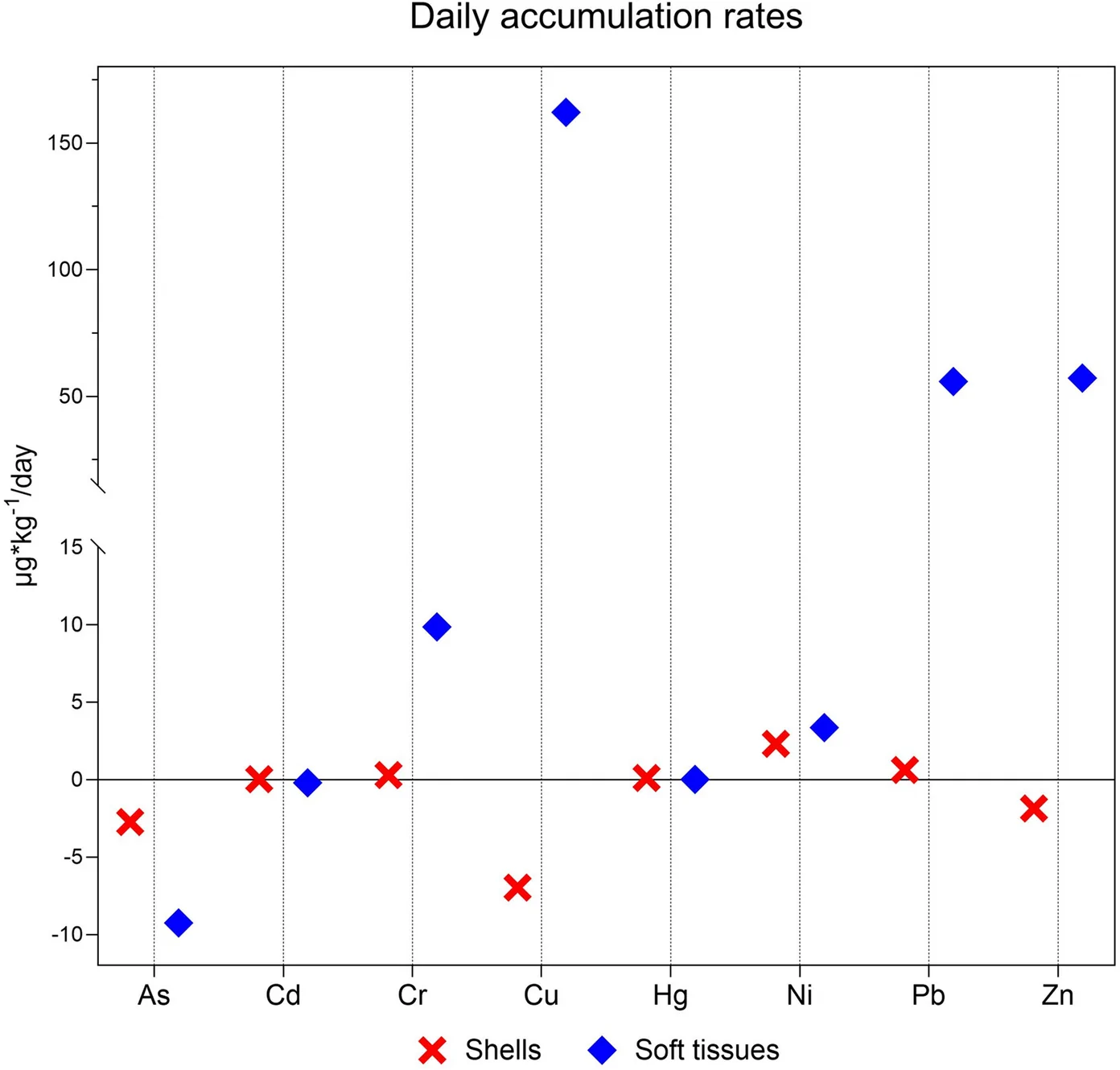
Daily net changes in whole-shell and soft-tissue element concentrations following 2.5 years of exposure, calculated as the difference between the mean concentrations in tissues and shells in organisms from the translocation site (Bagnera di Orta) and the donor site (Baveno), expressed in µg*kg^−1^/day

Daily net changes in element concentrations (Fig. 9) were more variable in soft tissues, with positive values for Cr, Cu, Pb, and Zn and a negative value for As. In contrast, whole-shell concentrations showed daily net changes generally close to zero for most elements, indicating only limited variation in average shell composition over the 904-day exposure period (Table S17).

## Discussion

The combined analysis of sediments, soft tissues and shells revealed a clear compartment-specific pattern of element distribution, reflecting the different temporal scales over which these matrices integrate environmental exposure. Sediments exhibited the strongest spatial differentiation among sites, followed by shells and, to a lesser extent, soft tissues. This pattern is consistent with the distinct ecological roles of the three compartments: sediments act as long-term integrators of environmental inputs, shells archive cumulative environmental exposure through biomineralization, whereas soft tissues primarily reflect recent, biologically mediated uptake (Protasowicki et al., 2008; Schöne & Krause, 2016). Sediments displayed the highest number of statistically significant differences among sites and the clearest separation between lakes in the PCA, confirming their sensitivity as indicators of long-term environmental conditions (Bonacina, 2001; Vignati et al., 2016). Despite the well documented history of severe industrial element contamination in Lake Orta, several elements occurred at higher concentrations in Lake Maggiore sediments. Although seemingly counterintuitive, this observation reflects a fundamental geochemical principle: present-day sediment chemistry is determined not only by historical contamination but also by catchment geology, lithogenic inputs, grain-size composition, sediment transport and post-depositional diagenetic processes (Horowitz, 1991; Kiralj et al., 2023; Luoma & Rainbow, 2008). The contrasting characteristics of the two catchments provide a plausible explanation for this pattern.

Lake Maggiore receives substantial detrital inputs from the Toce River, which drains one of the largest Alpine catchments in northern Italy. This basin is characterized by naturally element-rich lithologies and historical mining districts, delivering large quantities of fine mineral particles enriched in several trace elements (Baudo et al., 2002; Guerrieri et al., 2023; Marziali, 2025). In contrast, Lake Orta has a considerably smaller watershed and receives much lower lithogenic inputs. Arsenic provides a representative example, with concentrations in Baveno sediments exceeding those measured in Lake Orta while remaining fully consistent with the regional geochemical background previously reported for the Lake Maggiore basin (Baudo & Beltrami, 2001; Marziali, 2025). These findings therefore do not contradict the choice of Lake Maggiore as the reference site, which was selected because it has never experienced the severe anthropogenic element contamination that affected Lake Orta during much of the twentieth century. Importantly, higher total element concentrations in sediments should not be interpreted as evidence of greater biological uptake.

Bioaccumulation depends primarily on the bioavailable fraction rather than on total environmental concentrations, with bioavailability being controlled by element speciation, mineralogical composition, redox conditions, organic matter, particle size and physiological uptake mechanisms (Luoma & Rainbow, 2008; Rainbow, 2002; Tessier et al., 1984). Consequently, bulk sediment chemistry alone cannot reliably predict element accumulation in freshwater mussels. The transplantation experiment was specifically designed to address this limitation. By exposing mussels of known origin under natural environmental conditions for a defined period (904 days), it enabled the direct quantification of long-term changes in tissue and shell element concentrations while minimizing uncertainties associated with unknown exposure histories.

Soft tissues primarily reflected recent environmental exposure because of their relatively rapid element uptake and turnover, explaining their widespread use in biomonitoring programmes (Naimo, 1995; Rzymski et al., 2014). After prolonged exposure, translocated mussels from Bagnera exhibited tissue element concentrations remarkably similar to those of the resident population from Gozzano, indicating that local environmental conditions progressively become the dominant drivers of tissue element accumulation, whereas the influence of the donor population diminishes (Bervoets et al., 2004). The higher concentrations of Cu, Pb, Cr and Ni observed in Lake Orta, and of Na, Mn, Sr and As in Lake Maggiore, are consistent with differences in local geochemical conditions and exposure pathways (Lafrenière et al., 2025). However, the absence of a direct correspondence between sediment and tissue concentrations further supports the importance of element bioavailability and physiological regulation in determining biological uptake. Rather than reflecting bulk environmental concentrations, tissue element burdens integrate the combined effects of environmental availability, physiological regulation and species-specific element handling mechanisms.

Compared with soft tissues, shells exhibited a clearer spatial differentiation, reflecting their role as long-term archives of environmental exposure. Unlike soft tissues, which respond relatively rapidly to environmental changes, shell chemistry integrates element incorporation throughout shell growth by continuous biomineralization, preserving cumulative environmental signals over extended time scales while being less affected by short-term physiological fluctuations (Protasowicki et al., 2008; Schöne & Krause, 2016). This interpretation is supported by the PCA, in which shells from the transplanted population occupied an intermediate position between the donor population from Lake Maggiore and the resident population from Lake Orta, suggesting a progressive adjustment of shell composition towards local environmental conditions following transplantation. However, elemental incorporation into shell carbonate is regulated by a complex interplay among elemnt bioavailability, crystal chemistry, growth rate and physiological controls rather than being directly proportional to environmental concentrations (Besser et al., 1996; Roper & Hickey, 1995). Consequently, shell chemistry should be regarded as an integrated record of environmental exposure rather than as a direct proxy for dissolved or sediment element concentrations.

Bioconcentration factors (BCFs) further clarified the relationship between environmental availability and biological uptake. Essential elements involved in physiological processes were generally regulated within the organism and therefore did not necessarily reflect environmental concentrations. Sodium provides a clear example: its relatively low BCF values, despite comparable sediment concentrations, are consistent with the tight regulation of Na^+^ through branchial ion transport and Na^+^/K^+^ –ATPase activity in freshwater molluscs (Dietz & Branton, 1975; Griffith, 2017). Manganese displayed one of the most distinctive behaviours, exhibiting consistently high BCF values in all populations. This finding confirms the remarkable tendency of unionid mussels to selectively accumulate Mn, a phenomenon repeatedly reported in the literature but whose physiological basis remains only partially understood (Markich & Jeffree, 1994; Ravera, 2001). The affinity of freshwater bivalves for manganese is so pronounced that Mn concentration is routinely employed in gemmological investigations to distinguish freshwater pearls from marine pearls, freshwater pearls containing substantially higher Mn concentrations than their marine counterparts (Karampelas et al., 2019). The ecological significance of this characteristic was recognised more than sixty years ago, when Gaglione and Ravera (1964) reported the exceptional accumulation of the radionuclide ^54^Mn by Unio populations from Lake Maggiore. That pioneering study provided one of the earliest demonstrations of the extraordinary sensitivity of freshwater mussels as bioaccumulators of fallout-derived radionuclides, revealing the long-range transport of ^54^Mn released during atmospheric nuclear weapons testing in the Pacific Ocean (Gaglione & Ravera, 1964).

Collectively, these observations indicate that the preferential accumulation of Mn is an intrinsic biological characteristic of unionids rather than a simple consequence of environmental availability. Strontium also exhibited consistently high BCF values and is well known to substitute for calcium during shell biomineralization because of its similar ionic radius and chemical behaviour (Dobra et al., 2024; Markich & Jeffree, 1994). Barium displayed a comparable pattern, although its incorporation into aragonitic shells appears to be influenced by both environmental conditions and physiological regulation (Zhao et al., 2017).

Conversely, iron, cobalt and molybdenum showed relatively low BCF values despite their abundance in sediments, suggesting either limited bioavailability or efficient physiological regulation (Markich & Jeffree, 1994; Onishi & Nemoto, 2025). Zinc exhibited little spatial variation among populations, consistent with the tight homeostatic control of this essential element through metallothionein-mediated detoxification and storage mechanisms (Naimo, 1995; Marie et al., 2006).

By contrast, copper and lead displayed higher BCF values in Lake Orta than in Lake Maggiore despite comparable bulk sediment concentrations. This observation again emphasizes that total sediment concentrations alone are poor predictors of biological uptake.

Differences in sediment partitioning, element speciation, binding to iron and manganese oxides, and other geochemical factors may substantially alter element bioavailability and, consequently, accumulation by freshwater mussels (Nan et al., 2016; Tessier et al., 1984). Arsenic was the only element showing consistent spatial differences across sediments, soft tissues and shells, suggesting a strong link between its accumulation and site-related sediment-associated exposure, albeit over different time scales. Such behaviour agrees with the well-established geochemical control of arsenic mobility and speciation in freshwater ecosystems, and with the complex accumulation patterns previously described for freshwater bivalves (Rahman et al., 2012; Schaeffer et al., 2006; Smedley & Kinniburgh, 2002).

Mercury showed the opposite pattern: it was generally below detection limits in sediments and varied little among soft tissues, whereas shells exhibited clear spatial differences. This apparent discrepancy probably reflects the complex environmental cycling of mercury, for which total sediment concentrations often fail to represent biologically relevant exposure (Mancini et al., 2022). Because shell carbonate constitutes a metabolically inert structure that permanently incorporates trace elements during growth, shells may preserve historical exposure signals that are no longer detectable in soft tissues. This observation further supports the complementary value of shell chemistry for reconstructing long-term contamination histories.

The active transplantation experiment provided a unique opportunity to quantify temporal changes in element concentrations under environmentally realistic conditions while controlling one of the major sources of uncertainty affecting conventional field biomonitoring: the unknown exposure history of resident organisms. In natural populations, element concentrations measured in tissues or shells integrate exposure over the entire lifespan of the organism, making it difficult to estimate accumulation dynamics because neither the duration nor the environmental history of exposure is precisely known. By contrast, transplantation of mussels from a population with a known origin and a defined exposure period allowed the direct assessment of changes in elemental concentrations under natural environmental conditions. The daily net changes in element concentrations demonstrated that soft tissues responded much more rapidly to environmental change than shells. Soft tissues exhibited positive daily changes for Cr, Cu, Pb and Zn, indicating progressive adjustment to local environmental conditions following transplantation. By contrast, whole- shell concentrations changed only slightly over the same exposure period, confirming that shell chemistry integrates environmental information over considerably longer temporal scales through continuous biomineralization. It should be emphasized that the calculated daily net changes represent the average temporal variation in the elemental concentration of the analysed compartment over the 904-day exposure period rather than elemental incorporation rates into newly formed shell carbonate. Consequently, negative values do not imply shell dissolution or element release, but simply indicate that the average concentration measured after transplantation was lower than that of the donor population. For shells, this may result from the deposition of new carbonate containing lower concentrations of a given element than the previously formed shell, thereby modifying the average elemental composition of the whole shell while continuous shell growth proceeds. This interpretation is fully consistent with current concepts of shell geochemistry and sclerochronology, according to which shell chemistry reflects the combined effects of environmental exposure, biomineralization, growth dynamics and physiological regulation rather than a simple cumulative incorporation process (Luoma & Rainbow, 2008; Schöne & Krause, 2016). Although the present study focused on element bioaccumulation, the transplantation approach adopted here also provides a valuable framework for evaluating the potential of freshwater mussels in bioremediation. By quantifying temporal changes in element burdens under natural environmental conditions, this methodology represents an important step towards assessing the feasibility of using freshwater mussels as both biomonitors and nature-based remediation tools. Overall, our findings highlight the complementary value of sediments, soft tissues and shells for understanding element dynamics across different temporal scales and demonstrate the potential of active mussel transplantation as a robust approach for long- term biomonitoring in freshwater ecosystems.

## Conclusion

This study provides one of the first field-based assessments of element accumulation dynamics in transplanted freshwater mussels under natural environmental conditions. By integrating sediment chemistry with analyses of soft tissues and shells, we demonstrate that these compartments record environmental contamination over complementary temporal scales. Sediments provided the clearest picture of long-term geochemical conditions, soft tissues reflected recent biologically available exposure, whereas shells preserved a cumulative record of environmental history through biomineralization. Their combined analysis therefore offers a more comprehensive assessment of element contamination than any individual environmental matrix alone.

The transplantation experiment revealed a progressive convergence of element concentrations in the soft tissues of transplanted mussels towards those of the resident population, demonstrating that local environmental conditions are the primary drivers of long-term element accumulation. At the same time, our results confirm that bulk sediment chemistry alone cannot reliably predict biological uptake, which depends primarily on element bioavailability and physiological regulation. Integrating environmental chemistry with biological responses is therefore essential for a mechanistic understanding of contaminant dynamics in freshwater ecosystems. More broadly, this work highlights the advantages of active biomonitoring based on mussel transplantation. By controlling exposure time while maintaining natural environmental conditions, this approach can bridge the gap between laboratory bioaccumulation experiments and conventional field surveys, providing a robust framework for quantifying long-term element accumulation under realistic ecological conditions. Finally, the significant accumulation of several trace elements observed in transplanted mussels supports the growing evidence that freshwater mussels may serve not only as bioindicators but also as nature-based tools for ecosystem restoration and bioremediation. Future research should therefore quantify element sequestration rates under different environmental conditions and evaluate the ecosystem-scale effectiveness of freshwater mussels in integrated bioremediation strategies.

## Supplementary Information

Below is the link to the electronic supplementary material.

Supplementary file1 (DOCX 2112 KB)

## Data Availability

The data availability statement is accurate and correctly reflects the availability of the data supporting this study.
