# Supplementary material for: Trace element bioaccumulation in the freshwater mussel Unio elongatulus after long-term transplantation: implications for biomonitoring and nature-based remediation
Source: Environ Geochem Health. 2026 Aug 20;48(13):541. doi: 10.1007/s10653-026-03424-0 (PMC13490256; doi:10.1007/s10653-026-03424-0)
Supplement: Supplementary file 1 [file 10653_2026_3424_MOESM1_ESM.docx]

**SUPPLEMENTARY MATERIAL**

***Figure S1:*** *Map of the study area showing the three sampling sites: the donor population in Lake Maggiore (Baveno, green), the translocated population in Lake Orta (Bagnera di Orta, blue) and the resident population in Lake Orta (Gozzano, red).*

***Table S1:*** *Physicochemical characteristics of Lake Maggiore and Lake Orta during the study period. Values are reported as ranges when temporal variability was observed and were compiled from routine environmental monitoring data (CNR IRSA, Verbania).*

|  | **Lake Maggiore** | **Lake Orta** |
| --- | --- | --- |
| T (°C) | 4 - 26 | 5 - 27 |
| O_2_ (mg L^-1^) | 7.19 - 7.78 | 7.9 |
| pH | 7.24 - 7.38 | 6.9 |
| Conductivity (µS cm^-1^) | 147.6 - 151.6 | 102 |
| Alk (meq L^-1^) | 0.802 - 0.837 | 0.26 - 0.32 |
| Ca (mg L^-1^) | 21.8 - 22.8 | 9.3 - 13.2 |
| TP (µg L^-1^) | 11 - 13 | 4 - 6 |
| TN (mg L^-1^) | 0.83 - 0.90 | 1.1 - 1.6 |
| N-NH_4_ (µg L^-1^) | 2 - 6 | 3 - 4 |
| N-NO_3_ (µg L^-1^) | 771 - 793 | 1031 - 1512 |
| Cl (mg L^-1^) | 3.2 | 3.7 |
| SO_4_ (mg L^-1^) | 28.5 - 30 | 13.6 - 24.2 |
| Mg (mg L^-1^) | 3.6 - 3.8 | 1.6 |
| Na (mg L^-1^) | 2.9 | 4 |
| Si (mg L^-1^) | 1.62 - 1.85 | 0.2 - 1.5 |

***Table S2:*** *ICP-MS operating conditions.*

| ***Instrument parameters*** | ***Settings*** | |
| --- | --- | --- |
| RF Power (W) | | 1550 |
| Sampling Depth (mm) | | 9.0 |
| Plasma flow rate (L min^-1^) | | 15.0 |
| Carrier Gas (L min^-1^) | | Ar: 1.10 |
| Dilution Gas (L min^-1^) | | Ar: 0.10 |
| Tune mode | | Helium mode - high-energy helium mode (Se only) |
| He flow (ml min^-1^) | | 5.0 (10.0) |
| Peltier-cooler multipass condenser | | 2°C |
| Oxide/Doubly Charged Ratio | | ^140^Ce^16^O^+^/^140^Ce^+^: 0.339% |
|  | | ^140^Ce^2+^/^140^Ce: 1.416% |
| Internal standards | | ^6^Li, ^45^Sc, ^89^Y, ^115^In, ^159^Tb |
| Selected isotopes | | ^7^Li, ^51^V, ^52^Cr, ^59^Co, ^60^Ni, ^63^Cu, ^66^Zn, ^75^As, ^78^Se, ^85^Rb, ^95^Mo, ^111^Cd, ^121^Sb, ^137^Ba, ^201^Hg, ^208^Pb. |

***Table. S3:*** *ICP-OES operating conditions.*

| **Instrument parameters** | **Setting** |
| --- | --- |
| Read time (s) | 5 |
| Replicates | 3 |
| AVS | Yes (loop volume 1.5 mL) |
| Pump rate – uptake (mL/min) | 32.7 |
| Pump rate – Inject (mL/min) | 2.2 |
| Valve uptake delay (s) | 11.3 |
| Viewing modes | axial, radial(*) |
| Stabilisation time (s) | 15 (axial) – 40 (radial) |
| RF power (W) | 1500 |
| Aux flow rate (L min^-1^) | 1.00 |
| Plasma flow rate (L min^-1^) | 12.0 |
| Nebulizer flow rate (L min^-1^) | 0.70 |
| Elements (nm) | P (213.618) - Al (237.312) - Fe (238.204) - Mn (257.610) - Ca (393.366)* - Mg (280.270) - Na (589.592) - Sr (421.552) - K (766.491)* |
| Viewing height (mm) | 8 |
| Background correction | fitted |

***Table S4a:*** *Table S4a. Analytical calibration parameters for element determination by ICP-MS and ICP-OES, including method detection limits (MDLs), calibration ranges, and coefficients of determination (R²).*

| **ICP-MS** | **MDL**  **(mg kg^-1^)** | **calibration range**  **(μg L^-1^)** | **R^2^** |
| --- | --- | --- | --- |
| ^7^Li | 0.025 | 0-20 | 0.9999 |
| ^51^V | 0.0032 | 0-50 | 1.00000 |
| ^52^Cr | 0.011 | 0-500 | 1.00000 |
| ^59^Co | 0.007 | 0-50 | 1.00000 |
| ^60^Ni | 0.021 | 0-200 | 1.00000 |
| ^63^Cu | 0.015 | 0-5000 | 1.00000 |
| ^66^Zn | 0.032 | 0-20000 | 1.00000 |
| ^75^As | 0.0069 | 0-500 | 1.00000 |
| ^78^Se | 0.006 | 0-100 | 0.9999 |
| ^85^Rb | 0.0036 | 0-300 | 1.00000 |
| ^95^Mo | 0.006 | 0-20 | 1.00000 |
| ^111^Cd | 0.007 | 0-200 | 1.00000 |
| ^137^Ba | 3.0 |  | 1.00000 |
| ^121^Sb | 0.006 | 0-5 | 1.00000 |
| ^201^Hg | 0.025 | 0-5 | 0.9998 |
| ^208^Pb | 0.008 | 0-2000 | 1.00000 |
| **ICP-OES** | **MDL**  **(mg kg^-1^)** | **Dynamic range**  **(mg L^-1^)** |  |
| P (213.618) | 1.26 | 0-5 | 1.00000 |
| Al (237.312) | 0.25 | 0-100 | 0.9998 |
| Fe (238.204) | 0.011 | 0-200 | 1.00000 |
| Mn (257.610) | 0.010 | 0-2 | 1.00000 |
| Ca (393.366)* | 0.09 | 0-20 | 0.99997 |
| Mg (280.270) | 0.137 | 0-50 | 0.98995 |
| Na (589.592) | 0.639 | 0-2 | 0.99999 |
| Sr (421.552) | 0.019 | 0-1 | 1.00000 |
| K (766.491)* | 2.94 | 0-20 | 0.99999 |

*Table S4b. Analytical quality-control results for the IAEA-407 fish tissue and BCR-280R lake sediment reference materials, including recommended or certified concentrations, concentrations measured in the present study, recoveries, and relative standard deviations (RSDs).*

| **ICP-MS** | **Recommended values –**  **Fish Tissues**  **(mg kg^-1^)** | **Measured values–**  **Fish Tissues**  **(mg kg^-1^)** | **Recovery**  **(%)** | **RSD** | **Certified values –**  **Lake sediment**  **(mg kg^-1^)** | **Measured values–**  **Lake sediment**  **(mg kg^-1^)** | **Recovery**  **(%)** | **RSD** |
| --- | --- | --- | --- | --- | --- | --- | --- | --- |
| ^7^Li |  |  |  |  |  |  |  |  |
| ^51^V | 1.43 ± 0.20 | 1.44 ± 0.04 | 101 ± 2.8 | 2.8 |  |  |  |  |
| ^52^Cr | 0.73 ± 0.22 | 0.70 ± 0.02 | 96 ± 2.4 | 2.5 | 126 ± 7 | 120 ± 4 | 95 ± 3.3 | 3,5 |
| ^59^Co | 0.10 ± 0.02 | 0.095 ± 0.001 | 95 ± 0.9 | 0.9 | 16.8 ± 0.9 | 16.5 ± 0.8 | 98 ± 4.6 | 4,7 |
| ^60^Ni | 0.60 ± 0.18 | 0.52 ± 0,01 | 87 ± 1.0 | 1.1 | 69 ± 5 | 61 ± 2 | 89 ± 2.7 | 3,0 |
| ^63^Cu | 3.28 ± 0.40 | 2.95 ± 0.04 | 90 ± 1.1 | 1.2 | 53 ± 6 | 48 ± 3 | 91 ± 6.1 | 6,7 |
| ^66^Zn | 67.1 ± 3.8 | 69.11 ± 3.49 | 103 ± 5.2 | 5.0 | 224 ± 25 | 224 ± 6 | 100 ± 2.7 | 2,7 |
| ^75^As | 12.6 ± 1.2 | 12.3 ± 0.3 | 98 ± 2.1 | 2.1 | 33.4 ± 2.9 | 32 ± 2 | 96 ± 5.1 | 5,3 |
| ^78^Se | 2.83 ± 0.38 | 2.63 ± 0.13 | 93 ± 4.6 | 4.9 |  |  |  |  |
| ^85^Rb | 2.86 ± 0.40 | 2.57 ± 0.15 | 90 ± 5.4 | 6.0 |  |  |  |  |
| ^95^Mo |  |  |  |  |  |  |  |  |
| ^111^Cd | 0.189 ± 0.019 | 0.189 ± 0.002 | 100 ± 0.8 | 0.8 | 0.85 ± 0.10 | 0.84 ± 0.03 | 99 ± 3.9 | 3,9 |
| ^137^Ba |  |  |  |  |  |  |  |  |
| ^121^Sb |  |  |  |  |  |  |  |  |
| ^201^Hg | 0.222 ± 0.024 | 0.200 ± 0.012 | 90 ± 5.6 | 6.2 | 1.46 ± 0.20 | 1.36 ± 0.08 | 93 ± 5.4 | 5,8 |
| ^208^Pb | 0.12 ± 0.06 | 0.116 ± 0.003 | 97 ± 2.6 | 2.7 |  |  |  |  |
| **ICP-OES** | **Recommended values –**  **Fish Tissues**  **(mg kg^-1^)** | **Measured values–**  **Fish Tissues**  **(mg kg^-1^)** | **Recovery**  **(%)** | **RSD** | **Certified values –**  **Lake sediment**  **(mg kg^-1^)** | **Measured values–**  **Lake sediment**  **(mg kg^-1^)** | **Recovery**  **(%)** | **RSD** |
| P (213.618) |  |  |  |  |  |  |  |  |
| Al (237.312) | 13.8 ± 3.7 | 12.6 ± 0.8 | 91 ± 5.6 | 6.2 |  |  |  |  |
| Fe (238.204) | 146 ± 14 | 142 ± 3 | 97 ± 2.2 | 2.3 |  |  |  |  |
| Mn (257.610) | 3.52 ± 0.32 | 3.45 ± 0.05 | 98 ± 1.3 | 1.3 |  |  |  |  |
| Ca (393.366)* | 27000 ± 1800 | 24300 ± 1269 | 90 ± 4.7 | 5.2 |  |  |  |  |
| Mg (280.270) | 2720 ± 140 | 2638 ± 60 | 97 2.2 | 2.3 |  |  |  |  |
| Na (589.592) | 13100 ± 600 |  |  |  |  |  |  |  |
| Sr (421.552) | 130 ± 11 | 121 ± 2 | 93 ± 1.3 | 1.4 |  |  |  |  |
| K (766.491)* | 13100 ± 1200 | 11921 ± 734 | 91 ± 5.6 | 6.2 |  |  |  |  |

***Table S5****: Element concentration (mg•kg⁻¹) in sediments collected at the three sampling sites. Values are expressed as mean ± standard deviation calculated from five independent sediment samples collected and analysed separately at each site (n = 5).*

|  | **Gozzano** | | **Orta** | | **Baveno** | |
| --- | --- | --- | --- | --- | --- | --- |
| **Elements** | **Media** | **SD** | **Media** | **SD** | **Media** | **SD** |
| Al | 6983.39 | 1988.73 | 5972.58 | 391.11 | 10696.84 | 300.13 |
| As | 2.06 | 0.29 | 2.18 | 0.06 | 7.46 | 1.10 |
| Ba | 28.85 | 9.96 | 32.72 | 2.02 | 40.28 | 1.35 |
| Ca | 1430.93 | 386.72 | 1249.57 | 49.98 | 1734.65 | 156.88 |
| Cd | 0.66 | 0.08 | 0.66 | 0.02 | 0.73 | 0.03 |
| Co | 5.88 | 1.37 | 5.61 | 0.24 | 9.19 | 0.52 |
| Cr | 21.89 | 9.46 | 18.01 | 2.04 | 18.58 | 0.67 |
| Cu | 60.65 | 50.03 | 34.43 | 5.67 | 20.17 | 5.05 |
| Fe | 10595.18 | 3012.72 | 8719.96 | 669.61 | 16580.06 | 412.89 |
| Hg | <0.025 |  | <0.025 |  | 0.32 | 0.33 |
| K | 687.40 | 322.28 | 606.21 | 68.36 | 1907.04 | 64.00 |
| Li | 12.59 | 3.44 | 8.84 | 0.98 | 21.21 | 0.93 |
| Mg | 3370.86 | 856.16 | 2767.98 | 190.73 | 5189.17 | 124.46 |
| Mn | 113.20 | 31.40 | 89.23 | 5.55 | 158.72 | 3.17 |
| Mo | 1.30 | 0.08 | 1.32 | 0.03 | 1.44 | 0.03 |
| Na | 94.88 | 15.08 | 81.31 | 12.75 | 90.70 | 5.61 |
| Ni | 32.35 | 15.35 | 22.04 | 1.58 | 19.84 | 2.67 |
| P | 304.23 | 49.57 | 319.66 | 13.31 | 423.99 | 28.62 |
| Pb | 21.91 | 10.74 | 21.08 | 4.11 | 14.66 | 2.01 |
| Rb | 10.93 | 4.53 | 11.18 | 1.21 | 20.18 | 0.65 |
| Sb | 2.89 | 0.55 | 3.13 | 0.37 | 3.62 | 0.71 |
| Se | 0.40 | 0.12 | 0.41 | 0.18 | 0.82 | 0.07 |
| Sr | 5.51 | 1.71 | 5.21 | 0.25 | 11.06 | 0.82 |
| V | 17.00 | 4.43 | 13.54 | 1.02 | 21.34 | 0.24 |
| Zn | 99.31 | 58.74 | 62.58 | 9.23 | 64.21 | 12.86 |

***Table S6:*** *Elements concentration (mg•kg⁻¹) in soft tissues collected at the three sampling sites. Data is showed as Mean and SD.*

|  | **Gozzano** | | **Orta** | | **Baveno** | |
| --- | --- | --- | --- | --- | --- | --- |
| **Elements** | **Media** | **SD** | **Media** | **SD** | **Media** | **SD** |
| Al | 405.61 | 134.54 | 548.61 | 197.69 | 626.66 | 248.26 |
| As | 7.27 | 0.56 | 8.19 | 3.21 | 16.55 | 2.88 |
| Ca | 6632.86 | 3630.04 | 9048.40 | 1614.71 | 9986.17 | 2708.48 |
| Cd | 7.67 | 3.72 | 10.39 | 1.72 | 10.58 | 3.27 |
| Co | 1.15 | 0.34 | 1.72 | 0.60 | 1.95 | 0.56 |
| Cr | 15.54 | 6.16 | 12.90 | 4.02 | 3.99 | 1.10 |
| Cu | 113.38 | 32.80 | 154.83 | 90.58 | 8.28 | 1.33 |
| Fe | 3042.81 | 906.11 | 4036.61 | 1281.59 | 5307.22 | 1320.34 |
| Hg | 0.14 | 0.05 | 0.12 | 0.03 | 0.12 | 0.05 |
| K | 2287.16 | 286.92 | 2240.64 | 365.83 | 2458.84 | 318.45 |
| Li | 0.48 | 0.15 | 0.57 | 0.14 | 0.74 | 0.28 |
| Mg | 1573.46 | 283.74 | 1858.75 | 185.96 | 1713.10 | 376.67 |
| Mn | 2896.11 | 1705.72 | 5075.52 | 1463.51 | 8359.85 | 2810.16 |
| Mo | 0.50 | 0.13 | 0.53 | 0.10 | 0.54 | 0.10 |
| Na | 3893.41 | 984.48 | 4100.02 | 1026.25 | 5170.81 | 770.79 |
| Ni | 4.83 | 0.83 | 5.85 | 0.98 | 2.81 | 0.54 |
| Pb | 55.49 | 30.47 | 57.60 | 20.17 | 7.03 | 3.31 |
| Rb | 4.80 | 1.51 | 5.80 | 0.84 | 6.13 | 0.96 |
| Sb | 0.05 | 0.02 | 0.05 | 0.01 | 0.07 | 0.04 |
| Se | 2.93 | 0.50 | 2.74 | 0.51 | 2.96 | 0.21 |
| Sr | 132.45 | 59.13 | 302.26 | 86.40 | 554.15 | 189.53 |
| V | 0.80 | 0.23 | 0.94 | 0.30 | 1.20 | 0.37 |
| Zn | 425.90 | 188.07 | 488.62 | 93.27 | 436.88 | 145.59 |

***Table S7:*** *Elements concentration (mg•kg⁻¹) in shells collected at the three sampling sites. Data is showed as Mean and SD.*

|  | **Gozzano** | | **Orta** | | **Baveno** | |
| --- | --- | --- | --- | --- | --- | --- |
| **Elements** | **Media** | **SD** | **Media** | **SD** | **Media** | **SD** |
| Al | 20.81 | 19.79 | 18.59 | 8.95 | 28.21 | 12.17 |
| As | 0.16 | 0.07 | 0.46 | 0.18 | 2.94 | 1.45 |
| Ba | 59.75 | 9.72 | 38.32 | 4.00 | 42.18 | 7.63 |
| Ca | 388022.38 | 30923.33 | 361311.64 | 6870.45 | 345011.98 | 95973.50 |
| Cd | 0.06 | 0.07 | 0.06 | 0.03 | 0.03 | 0.02 |
| Co | 0.06 | 0.03 | 0.08 | 0.03 | 0.09 | 0.03 |
| Cr | 1.12 | 0.17 | 1.00 | 0.11 | 0.74 | 0.06 |
| Cu | 31.04 | 47.29 | 9.60 | 4.79 | 15.90 | 10.84 |
| Fe | 62.06 | 36.72 | 170.30 | 72.90 | 313.99 | 121.54 |
| Hg | 0.47 | 0.13 | 0.21 | 0.05 | 0.11 | 0.02 |
| K | 49.17 | 7.70 | 76.80 | 30.64 | 59.60 | 24.86 |
| Li | 0.34 | 0.11 | 0.24 | 0.13 | 0.19 | 0.13 |
| Mg | 23.99 | 12.53 | 32.79 | 11.68 | 30.23 | 11.56 |
| Mn | 161.50 | 46.46 | 215.82 | 58.94 | 260.77 | 62.71 |
| Mo | 0.04 | 0.01 | 0.04 | 0.01 | 0.07 | 0.05 |
| Na | 1947.98 | 149.43 | 1657.90 | 57.07 | 1807.12 | 153.46 |
| Ni | 1.10 | 0.91 | 2.37 | 0.61 | 0.28 | 0.15 |
| P | 89.18 | 17.96 | 104.37 | 13.22 | 108.24 | 17.70 |
| Pb | 1.74 | 2.00 | 1.54 | 0.41 | 0.97 | 0.68 |
| Rb | 0.16 | 0.09 | 0.04 | 0.04 | 0.03 | 0.03 |
| Sb | 0.16 | 0.13 | 0.31 | 0.35 | 1.43 | 3.53 |
| Se | 0.16 | 0.13 | 0.15 | 0.10 | 0.14 | 0.07 |
| Sr | 392.62 | 37.00 | 625.76 | 81.67 | 909.80 | 150.58 |
| V | 0.10 | 0.07 | 0.11 | 0.04 | 0.16 | 0.06 |
| Zn | 22.99 | 34.80 | 7.98 | 5.67 | 9.65 | 8.34 |

***Table S8:*** *P-values obtained from the statistical analysis of elment concentrations in sediment, soft tissue and shell, between the three sampling sites; one-way ANOVA or non-parametric Kruskal-Wallis test were employed. Significant p-values are highlighted (* = p < 0.05; ** = p < 0.01; *** = p < 0.001; **** = p < 0.0001; ns = not significant; nm = not measured; bdl = below detection limit).*

| **Elements** | **Sediment** | **Sign.** | **Tissue** | **Sign.** | **Shell** | **Sign.** |
| --- | --- | --- | --- | --- | --- | --- |
| Al | 0.0001 | **** | 0.1823 | NS | 0.268 | NS |
| As | 0.0001 | **** | 0.0001 | **** | 0.0001 | **** |
| Ba | 0.0287 | * | - | NM | 0.0001 | **** |
| Ca | 0.0312 | * | 0.2643 | NS | 0.2427 | NS |
| Cd | 0.1068 | NS | 0.1414 | NS | 0.2021 | NS |
| Co | 0.0001 | **** | 0.0130 | * | 0.1208 | NS |
| Cr | 0.5894 | NS | 0.0001 | **** | 0.0001 | **** |
| Cu | 0.0536 | NS | 0.0001 | **** | 0.3917 | NS |
| Fe | 0.0006 | *** | 0.0117 | * | 0.0001 | **** |
| Hg | - | BDL | 0.6179 | NS | 0.0001 | **** |
| K | 0.0001 | **** | 0.3397 | NS | 0.1169 | NS |
| Li | 0.0001 | **** | 0.0862 | NS | 0.0882 | NS |
| Mg | 0.0004 | *** | 0.1175 | NS | 0.5072 | NS |
| Mn | 0.0002 | *** | 0.0005 | *** | 0.0085 | ** |
| Mo | 0.0029 | ** | 0.6684 | NS | 0.0052 | ** |
| Na | 0.2214 | NS | 0.0155 | * | 0.0017 | ** |
| Ni | 0.0886 | NS | 0.0001 | **** | 0.0003 | *** |
| P | 0.0002 | *** | - | NM | 0.0873 | NS |
| Pb | 0.0364 | * | 0.0002 | *** | 0.0406 | * |
| Rb | 0.0005 | *** | 0.0664 | NS | 0.0003 | *** |
| Sb | 0.1646 | NS | 0.2154 | NS | 0.1931 | NS |
| Se | 0.0008 | *** | 0.4225 | NS | 0.7794 | NS |
| Sr | 0.0001 | **** | 0.0001 | **** | 0.0001 | **** |
| V | 0.0019 | ** | 0.0736 | NS | 0.0715 | NS |
| Zn | 0.2309 | NS | 0.608 | NS | 0.3785 | NS |

***Table S9:*** *Results of one-way ANOVA or Kruskal–Wallis tests testing differences in element concentrations in sediment among sites. For elements showing a significant global effect (p < 0.05), Tukey HSD or Dunn post hoc tests were applied. Significant pairwise comparisons are reported as site pairs. Site abbreviations: B = Baveno; G = Gozzano; O = Orta.*

| **Elements** | **Test** | **Statistic** | **p (global)** | **Post hoc test** | **Sig. comp.** | **Post hoc p** |
| --- | --- | --- | --- | --- | --- | --- |
| Al | ANOVA | F = 22.11 | <0.0001 | Tukey | G-B; O-B | 0.0009; 0.0001 |
| As | ANOVA | F = 109.4 | <0.0001 | Tukey | G-B; O-B | <0.0001; <0.0001 |
| Ba | ANOVA | F = 4.845 | 0.0287 | Tukey | G-B | 0.0249 |
| Ca | KW | H = 6.5 | 0.0312 | Dunn | O-B | 0.04 |
| Cd | ANOVA | F = 2.711 | 0.1068 | - | - | - |
| Co | ANOVA | F = 22.29 | <0.0001 | Tukey | G-B; O-B | 0.0003; 0.0002 |
| Cr | ANOVA | F = 0.5526 | 0.5894 | - | - | - |
| Cu | ANOVA | F = 3.771 | 0.0536 | - | - | - |
| Fe | KW | H = 10.22 | 0.0006 | Dunn | O-B | 0.0056 |
| Hg | - | - | - | - | - | - |
| K | ANOVA | F = 70.72 | <0.0001 | Tukey | G-B; O-B | <0.0001; <0.0001 |
| Li | ANOVA | F = 44.28 | <0.0001 | Tukey | G-B; O-B | <0.0001; <0.0001 |
| Mg | KW | H = 10.5 | 0.0004 | Dunn | O-B | 0.0044 |
| Mn | ANOVA | F = 18.2 | 0.0002 | Tukey | G-B; O-B | 0.0056; 0.0002 |
| Mo | KW | H = 9.380 | 0.0029 | Dunn | G-B; O-B | 0.0216; 0.0267 |
| Na | ANOVA | F = 1.714 | 0.2214 | - | - | - |
| Ni | KW | H = 4.820 | 0.0886 | - | - | - |
| P | ANOVA | F = 18.44 | 0.0002 | Tukey | G-B; O-B | 0.0003; 0.0010 |
| Pb | KW | H = 6.18 | 0.0364 | Dunn | - | - |
| Rb | ANOVA | F = 15.24 | 0.0005 | Tukey | G-B; O-B | 0.0009; 0.0016 |
| Sb | KW | H = 3.660 | 0.1646 | - | - | - |
| Se | ANOVA | F = 13.62 | 0.0008 | Tukey | G-B; O-B | 0.0018; 0.0020 |
| Sr | ANOVA | F = 34.33 | <0.0001 | Tukey | G-B; O-B | <0.0001; <0.0001 |
| V | ANOVA | F = 11.01 | 0.0019 | Tukey | O-B | 0.0014 |
| Zn | ANOVA | F = 1.66 | 0.2309 | - | - | - |

***Table S10:*** *Loadings of sediment element concentrations on the first two principal components.*

| **Var** | **PC1** | **PC2** |
| --- | --- | --- |
| Al | 0.992 | 0.010 |
| As | 0.885 | -0.415 |
| Ba | 0.869 | 0.226 |
| Ca | 0.917 | 0.309 |
| Cd | 0.843 | 0.233 |
| Co | 0.992 | -0.078 |
| Cr | 0.376 | 0.914 |
| Cu | 0.062 | 0.980 |
| Fe | 0.988 | -0.013 |
| Hg | 0.601 | -0.259 |
| K | 0.961 | -0.224 |
| Li | 0.965 | -0.071 |
| Mg | 0.984 | -0.031 |
| Mn | 0.972 | 0.096 |
| Mo | 0.932 | -0.006 |
| Ni | 0.130 | 0.970 |
| P | 0.923 | -0.150 |
| Pb | -0.068 | 0.881 |
| Rb | 0.981 | -0.032 |
| Sb | 0.439 | -0.347 |
| Se | 0.798 | -0.356 |
| Sr | 0.971 | -0.202 |
| V | 0.943 | 0.240 |
| Zn | 0.272 | 0.949 |
| Eigenvalues | 15.8 | 5.3 |
| Proportion of variance (%) | 65.63% | 22.06% |
| Cumulative proportion (%) | 65.63% | 87.70% |

***Table S11:*** *Results of one-way ANOVA or Kruskal–Wallis tests testing differences in elment concentrations in soft tissues among sites. For elements showing a significant global effect (p < 0.05), Tukey HSD or Dunn post hoc tests were applied. Only significant pairwise comparisons are reported. Site abbreviations: B = Baveno; G = Gozzano; O = Orta.*

| **Elements** | **Test** | **Statistic** | **p (global)** | **Post hoc test** | **Sig. comp.** | **Post hoc p** |
| --- | --- | --- | --- | --- | --- | --- |
| Al | ANOVA | F = 1.848 | 0.1823 | - | - | - |
| As | ANOVA | F = 31.47 | <0.0001 | Tukey | G-B; O-B | <0.0001; <0.0001 |
| Ba | - | - | - | - | - | - |
| Ca | KW | H = 2.662 | 0.2643 | - | - | - |
| Cd | ANOVA | F = 2.151 | 0.1414 | - | - | - |
| Co | ANOVA | F = 5.378 | 0.0130 | Tukey | G-B | 0.0097 |
| Cr | ANOVA | F = 35.89 | <0.0001 | Tukey | G-B; O-B | <0.0001; <0.0001 |
| Cu | ANOVA | F = 103.8 | <0.0001 | Tukey | G-B; O-B | <0.0001; <0.0001 |
| Fe | KW | H = 8.889 | 0.0117 | Dunn | G-B | 0.0122 |
| Hg | ANOVA | F = 0.492 | 0.6179 | - | - | - |
| K | ANOVA | F = 1.137 | 0.3397 | - | - | - |
| Li | ANOVA | F = 2.76 | 0.0862 | - | - | - |
| Mg | KW | H = 4.282 | 0.1175 | - | - | - |
| Mn | ANOVA | F = 11.25 | 0.0005 | Tukey | G-B; O-B | 0.0006; 0.018 |
| Mo | KW | H = 0.805 | 0.6684 | - | - | - |
| Na | ANOVA | F = 5.118 | 0.0155 | Tukey | G-B; O-B | 0.0352; 0.0498 |
| Ni | KW | H = 17.90 | 0.0001 | Dunn | G-B; O-B | 0.0219; 0.0002 |
| P | - | - | - | - | - | - |
| Pb | KW | H = 17.28 | 0.0002 | Dunn | G-B; O-B | 0.0047; 0.001 |
| Rb | ANOVA | F = 3.094 | 0.0664 | - | - | - |
| Sb | KW | H = 3.071 | 0.2154 | - | - | - |
| Se | ANOVA | F = 0.897 | 0.4225 | - | - | - |
| Sr | ANOVA | F = 16.39 | <0.0001 | Tukey | G-B; O-B | <0.0001; 0.0046 |
| V | ANOVA | F = 2.962 | 0.0736 | - | - | - |
| Zn | ANOVA | F = 0.509 | 0.608 | - | - | - |

***Table S12:*** *Loadings of soft tissues element concentrations on the first two principal components.*

| **Var** | **PC1** | **PC2** |
| --- | --- | --- |
| As | -0.917 | -0.026 |
| Co | -0.620 | 0.745 |
| Cr | 0.755 | 0.354 |
| Cu | 0.750 | 0.527 |
| Fe | -0.773 | 0.508 |
| Na | -0.585 | 0.387 |
| Ni | 0.737 | 0.548 |
| Mn | -0.853 | 0.285 |
| Pb | 0.794 | 0.538 |
| Sr | -0.892 | 0.217 |
| Eigenvalues | 5.996 | 2.085 |
| Proportion of variance (%) | 59.96% | 20.85% |
| Cumulative proportion (%) | 59.96% | 80.81% |

***Table S13:*** *Bioconcentration factors, calculated for each element as the ratio between sediment concentrations and those measured in each compartment.*

| **Elements** | **Tissue** | | | **Shell** | | |
| --- | --- | --- | --- | --- | --- | --- |
|  | **Gozzano** | **Orta** | **Baveno** | **Gozzano** | **Orta** | **Baveno** |
| Al | 0.058 | 0.092 | 0.059 | 0.003 | 0.003 | 0.003 |
| As | 3.527 | 3.759 | 2.218 | 0.075 | 0.213 | 0.394 |
| Ba | 2.071 | 1.171 | 1.047 | - | - | - |
| Ca | 4.635 | 7.241 | 5.757 | 271.169 | 289.148 | 198.894 |
| Cd | 11.543 | 15.755 | 14.489 | 0.088 | 0.084 | 0.043 |
| Co | 0.196 | 0.307 | 0.212 | 0.011 | 0.015 | 0.010 |
| Cr | 0.710 | 0.716 | 0.215 | 0.051 | 0.056 | 0.040 |
| Cu | 1.869 | 4.497 | 0.410 | 0.512 | 0.279 | 0.788 |
| Fe | 0.287 | 0.463 | 0.320 | 0.006 | 0.020 | 0.019 |
| K | 3.327 | 3.696 | 1.289 | 0.072 | 0.127 | 0.031 |
| Li | 0.038 | 0.065 | 0.035 | 0.027 | 0.027 | 0.009 |
| Mg | 0.467 | 0.672 | 0.330 | 0.007 | 0.012 | 0.006 |
| Mn | 25.585 | 56.884 | 52.671 | 1.427 | 2.419 | 1.643 |
| Mo | 0.388 | 0.405 | 0.374 | 0.032 | 0.032 | 0.049 |
| Na | 41.034 | 50.423 | 57.011 | 20.530 | 20.389 | 19.924 |
| Ni | 0.149 | 0.265 | 0.142 | 0.034 | 0.108 | 0.014 |
| P | 0.293 | 0.326 | 0.255 | - | - | - |
| Pb | 2.533 | 2.732 | 0.480 | 0.079 | 0.073 | 0.066 |
| Rb | 0.439 | 0.519 | 0.304 | 0.015 | 0.004 | 0.002 |
| Sb | 0.018 | 0.015 | 0.019 | 0.055 | 0.099 | 0.394 |
| Se | 7.418 | 6.732 | 3.594 | 0.406 | 0.365 | 0.173 |
| Sr | 24.049 | 58.012 | 50.099 | 71.289 | 120.101 | 82.253 |
| V | 0.047 | 0.069 | 0.056 | 0.006 | 0.008 | 0.008 |
| Zn | 4.288 | 7.807 | 6.804 | 0.231 | 0.127 | 0.150 |

***Table S14:*** *Results of one-way ANOVA or Kruskal–Wallis tests testing differences in element concentrations in shells among sites. For elements showing a significant global effect (p < 0.05), Tukey HSD or Dunn post hoc tests were applied. Only significant pairwise comparisons are reported. Site abbreviations: B = Baveno; G = Gozzano; O = Orta.*

| **Elements** | **Test** | **Statistic** | **p (global)** | **Post hoc test** | **Sig. comp.** | **Post hoc p** |
| --- | --- | --- | --- | --- | --- | --- |
| Al | ANOVA | F = 1.395 | 0.268 | - | - | - |
| As | ANOVA | F = 21.75 | <0.0001 | Tukey | G-B; O-B | <0.0001; <0.0001 |
| Ba | ANOVA | F = 16.45 | <0.0001 | Tukey | G-O; G-B | <0.0001; 0.0002 |
| Ca | KW | H = 2.832 | 0.2427 | - | - | - |
| Cd | ANOVA | F = 1.715 | 0.2021 | - | - | - |
| Co | ANOVA | F = 2.320 | 0.1208 | - | - | - |
| Cr | ANOVA | F = 30.85 | <0.0001 | Tukey | G-B; O-B | <0.0001; <0.0001 |
| Cu | ANOVA | F = 0.976 | 0.3917 | - | - | - |
| Fe | KW | H = 17.7 | 0.0001 | Dunn | G-B | <0.0001 |
| Hg | ANOVA | F = 80.43 | <0.0001 | Tukey | G-O; G-B; O-B | <0.0001; <0.0001; <0.0001 |
| K | ANOVA | F = 2.36 | 0.1169 | - | - | - |
| Li | ANOVA | F = 2.704 | 0.0882 | - | - | - |
| Mg | KW | H = 1.358 | 0.5072 | - | - | - |
| Mn | ANOVA | F = 5.9 | 0.0085 | Tukey | G-B | 0.0067 |
| Mo | KW | H = 10.50 | 0.0052 | Dunn | G-B; O-B | 0.0366; 0.0140 |
| Na | ANOVA | F = 8.547 | 0.0017 | Tukey | G-O | 0.0012 |
| Ni | KW | H = 16.56 | 0.0003 | Dunn | O-B | 0.0001 |
| P | ANOVA | F = 2.717 | 0.0873 | - | - | - |
| Pb | KW | H = 6.406 | 0.0406 | Dunn | O-B | 0.0342 |
| Rb | ANOVA | F = 11.98 | 0.0003 | Tukey | G-O; G-B | 0.0020; 0.0003 |
| Sb | KW | H = 3.289 | 0.1931 | - | - | - |
| Se | ANOVA | F = 0.252 | 0.7794 | - | - | - |
| Sr | ANOVA | F = 43.23 | <0.0001 | Tukey | G-O; G-B; O-B | 0.0028; <0.0001; <0.0001 |
| V | ANOVA | F = 2.966 | 0.0715 | - | - | - |
| Zn | ANOVA | F = 1.014 | 0.3785 | - | - | - |

***Table S15:*** *Loadings of shell elements concentrations on the first two principal components.*

| **Var** | **PC1** | **PC2** |
| --- | --- | --- |
| As | 0.845 | 0.391 |
| Ba | -0.513 | 0.684 |
| Cr | -0.828 | 0.131 |
| Fe | 0.850 | 0.310 |
| Hg | -0.893 | 0.295 |
| Mn | 0.648 | 0.410 |
| Mo | 0.468 | 0.523 |
| Na | -0.221 | 0.615 |
| Ni | -0.447 | -0.298 |
| Pb | -0.417 | 0.480 |
| Rb | -0.706 | 0.488 |
| Sr | 0.884 | 0.185 |
| Eigenvalues | 5.523 | 2.233 |
| Proportion of variance (%) | 46.03% | 18.61% |
| Cumulative proportion (%) | 46.03% | 64.64% |

***Table S16:*** *Relative abundance of elements in sediments, soft tissues, and shells at the three sampling sites, expressed as decreasing order of mean concentrations.*

| **Compartment** | **Site** | **Sequences** |
| --- | --- | --- |
| **Sediment** | Gozzano | Fe>Al>Mg>Ca>K>P>Mn>Zn>Na>Cu>Ni>Ba>Pb>Cr>V>Li>  Rb>Co>Sr>Sb>As>Mo>Cd>Se>Hg |
|  | Orta | Fe>Al>Mg>Ca>K>P>Mn>Na>Zn>Cu>Ba>Ni>Pb>Cr>V>Rb>  Li>Co>Sr>Sb>As>Mo>Cd>Se>Hg |
|  | Baveno | Fe>Al>Mg>K>Ca>P>Mn>Na>Zn>Ba>V>Li>Rb>Cu>Ni>Cr>  Pb>Sr>Co>As>Sb>Mo>Se>Cd>Hg |
| **Soft tissues** | Gozzano | Ca>Na>Fe>Mn>K>Mg>Zn>Al>Sr>Cu>Pb>Cr>Cd>As>Ni>  Rb>Se>Co>V>Mo>Li>Hg>Sb |
|  | Orta | Ca>Mn>Na>Fe>K>Mg>Al>Zn>Sr>Cu>Pb>Cr>Cd>As>Ni>  Rb>Se>Co>V>Li>Mo>Hg>Sb |
|  | Baveno | Ca>Mn>Fe>Na>K>Mg>Al>Sr>Zn>As>Cd>Cu>Pb>Rb>Cr>  Se>Ni>Co>V>Li>Mo>Hg>Sb |
| **Shell** | Gozzano | Ca>Na>Sr>Mn>P>Fe>Ba>K>Cu>Mg>Zn>Al>Pb>Cr>Ni>  Hg>Li>Rb>Se>Sb>As>V>Co>Cd>Mo |
|  | Orta | Ca>Na>Sr>Mn>Fe>P>K>Ba>Mg>Al>Cu>Zn>Ni>Pb>Cr>  As>Sb>Li>Hg>Se>V>Co>Cd>Mo>Rb |
|  | Baveno | Ca>Na>Sr>Fe>Mn>P>K>Ba>Mg>Al>Cu>Zn>As>Sb>Pb>  Cr>Ni>Li>V>Se>Hg>Co>Mo>Rb>Cd |

***Table S17:*** *Daily accumulation rates of elements in shells and soft tissues, calculated as the difference between the mean concentrations in tissues and shells in organisms from the translocation site (Bagnera di Orta) and the donor site (Baveno), expressed in µg*kg^-1^/day.*

| **Elements** | **Shells** | **Soft tissues** |
| --- | --- | --- |
| As | -2.74 | -9.25 |
| Cd | 0.03 | -0.20 |
| Cr | 0.29 | 9.86 |
| Cu | -6.97 | 162.11 |
| Hg | 0.11 | 0.01 |
| Ni | 2.32 | 3.36 |
| Pb | 0.63 | 55.93 |
| Zn | -1.86 | 57.23 |
